# Optimized network based natural language processing approach to reveal disease comorbidities in COVID-19

**DOI:** 10.1038/s41598-024-52819-5

**Published:** 2024-01-28

**Authors:** Emre Taylan Duman, Gizem Tuna, Enes Ak, Gülben Avsar, Pinar Pir

**Affiliations:** 1 Department of Bioengineering, Gebze Technical University, Kocaeli, Turkey; 2 NGS-Core Unit for Integrative Genomics, Institute of Pathology, University Medical Center Göttingen, Göttingen, Germany; 3 Department of Molecular Biology, Gebze Technical University, Kocaeli, Turkey

**Keywords:** Machine learning, Computational biology and bioinformatics, Predictive medicine, Comorbidities

## Abstract

A novel virus emerged from Wuhan, China, at the end of 2019 and quickly evolved into a pandemic, significantly impacting various industries, especially healthcare. One critical lesson from COVID-19 is the importance of understanding and predicting underlying comorbidities to better prioritize care and pharmacological therapies. Factors like age, race, and comorbidity history are crucial in determining disease mortality. While clinical data from hospitals and cohorts have led to the identification of these comorbidities, traditional approaches often lack a mechanistic understanding of the connections between them. In response, we utilized a deep learning approach to integrate COVID-19 data with data from other diseases, aiming to detect comorbidities with mechanistic insights. Our modified algorithm in the mpDisNet package, based on word-embedding deep learning techniques, incorporates miRNA expression profiles from SARS-CoV-2 infected cell lines and their target transcription factors. This approach is aligned with the emerging field of network medicine, which seeks to define diseases based on distinct pathomechanisms rather than just phenotypes. The main aim is discovery of possible unknown comorbidities by connecting the diseases by their miRNA mediated regulatory interactions. The algorithm can predict the majority of COVID-19's known comorbidities, as well as several diseases that have yet to be discovered to be comorbid with COVID-19. These potentially comorbid diseases should be investigated further to raise awareness and prevention, as well as informing the comorbidity research for the next possible outbreak.

## Introduction

SARS-CoV-2 virus could infect many types of human cells, and also has the ability of rapid evolution. Even though numerous strains of the virus have been identified^1,2^, the virus's infection process in all strains relies on the ACE2 receptor protein binding to spike proteins as reported by the early investigations of the infection^3^. The vascular effects of the ACE/ACE2 balance were also found to be influenced by viral binding, leading to several complications. These effects have serious consequences in patients with diseases that cause an inbalance in ACEs ratio, such as diabetes, heart disease, and blood tension-related disorders. This shared mechanism makes COVID-19 highly comorbid and rare comorbidities with unknown backgrounds could be still undiscovered. In this paper, possible comorbidities of COVID-19 examined by a network-based representation of miRNA-gene and disease interactions to reveal possibly co-altered mechanisms.

Mendelian and complex (multifactorial) are the two most common classifications for diseases. Mendelian disorders are caused by a single gene mutation and may be identified by their inheritance patterns. Complex diseases also have a genetic component but exhibit complex genetic patterns, in addition, environmental parameters play a larger role in complex diseases than in the Mendelian diseases^4^. Next-generation sequencing applications have improved the quality and coverage of acquired data, by combining data from diverse omics techniques, it is possible to gain a deeper knowledge of both mendelian and complex disorders. Identifying multiple molecular and mechanistic signatures of etiology of the diseases via multi-scale approaches can offer us a more comprehensive understanding of mechanisms and may allow us to construct accurate disease linkages^5^. For example, integration of clinic next-generation sequencing and the human protein–protein interaction network (PPI)^6^ improved our understanding of the exact mechanisms of diseases^7,8^. Investigation of multiple layers, including gene regulation and miRNA profiles has the power to provide even more details on indirect effects of disease-associated genes.

Jin et al. proposed a meta-path-based disease network identification method, which is a network-based approach for discovering disease comorbidities by incorporating miRNA-mediated network structure into the word2vec method^8^. MpDisNet uses four different biological networks to create a network structure that connects diseases that may be comorbid: disease-miRNA (miR2Disease), miRNA-gene^9^, disease-gene^10^, and human PPI. The word2vec is a Natural Language Processing (NLP) technique, that basically calculates word representations based on semantic similarity, which assumes that similar words have tendency to be in similar sentences. Using MpDisNET, disease similarities are obtained by representation of disease-miRNA-gene–gene-miRNA-disease meta-path as sentences. The algorithm begins with a random walk that selects the first disease from the set. The selection of the disease continues with one random selection of the miRNA that is possibly related with the previously selected disease and proceeds with the possible gene list that is related with the miRNA^8^. As a result, algorithm derives word-like representations to train a word embedding algorithm which leads to vector representations of each disease. Similarities of these vectors are used as a metric for comorbidity between diseases.

The focus of this work is on fine-tuning the performance of miRNA-mediated disease interaction networks in detection of comorbidities. We propose two modifications to improve the algorithm. First, by removing cancer dominance from the training data, the performance of the present algorithm can be improved. Second, inclusion of regulatory information in the models also leads to better performance. To do so, we updated the algorithm by replacing the disease-miRNA-gene interaction network by a disease-miRNA-TF based network, hence by incorporating regulatory information into the system. We have also gathered a larger set of comorbidity data to improve the accuracy of performance measurements. Using the improved algorithm, miRNA and gene regulatory networks, comorbidities of COVID-19 was identified and validated using clinical data. This work shows how deep-learning technologies can be used to uncover comorbidities of a novel disease such as COVID-19. This method can be further improved to increase its predictive value by incorporating multi-omic data, expanding the network size, and using alternative word-embedding approaches.

## Methods

### COVID-miRNA data implementation

The non-coding RNA expression data generated by Wyler et al. (GSE148729) was used to develop the disease-disease interaction network for COVID-19 as a case study. Wyler et al. used lung-based carcinomic cell lines Calu-3, Caco-2, and H1299 to gather non-coding RNA, bulk RNA, and scRNA-seq data inSARS-CoV-2 infection. These cell lines activated their viral RNA receptor gene expressions with the exception of Caco-2. 11 miRNAs that have been identified to be upregulated in this dataset in SARS-COV-2 infection, these miRNAs were integrated to the default miRNA list in the mpDisNet algorithm (Supplementary Document: miRNA_list.pdf).

### Integration of TF-TF regulation network

One of the goals of this work was to improve prediction power of the algorithm by using network of direct regulation mechanism instead of a protein–protein interaction dataset, which does not particularly capture regulatory interactions between two proteins. PPI data from Intact, PINA, and HPRD were included by default in mpDisNet, which included all known protein interactions^6,11,12^. Even though this strategy enhances randomness in the algorithm, it may lead to the dominance of widely investigated diseases with many associated miRNAs in the model training, which produces large number of false positive discoveries and reduces the likelihood of clinically observed comorbidities being discovered. In this work, experimentally confirmed regulatory connections between transcription factors and genes have been gathered to reduce this dominance effect by increasing specificity of the gene–gene interactions. Use of TF interactions instead of human PPI, would reduce the effect of heterogenic distribution of the disease frequencies by removing non-regulatory gene–gene interactions. The TF-TF list was generated by integration of the data from 5 resources as shown in Table 1.

**Table 1:** Number of transcription factors and their interactions in the corresponding databases implemented into the miRNA mediated disease-disease interaction network.

Database	Number of TFs	Number of interactions
Marbach et. al.^12^	643	13,05,782
ChEA^6^	199	3,86,625
ENCODE^13^	181	16,51,393
TRANSFAC^14^	201	1,00,560
TRRUST v2^15^	795	8,428
Total	1168	31,25,927

We were able to collect 1168 transcription factors that take part in over 3 million experimentally validated TF-gene interaction pairs at the end of the integration process. Marbach et al.^13^ has one of the most comprehensive TF-Gene interaction datasets with experimental evidence in the literature. Also, 3 datasets with same feature (ChEA, ENCODE and TRANSFAC) collected from Harmonizome^14^ database. Finally, when TRRUST v2 was integrated, we obtained our final TF-Gene interaction network with removal of identical interactions. We hypothesize that this data can point to a more direct genetic link between two potentially comorbid disorders. Transcription factors that regulate another transcription factor are chosen from the entire list, yielding 28.000 TF-TF interaction pairs (Supplementary Table: TF-TF_list.xlsx).

Training has been performed for four different setups. First (Reproduced), is the original meta-path which is exactly same setup used in the original mpDisNet implementation (Disease1-miRNA1-Gene1-Gene2-miRNA2-Disease2). A second trial has been made on modified disease list which has cancer-related diseases removed and COVID-19 added (Covid_cancerless) with the same meta-path as the original data. Third and fourth setups use modified meta-path based on the TF-TF regulation network with and without cancer related terms (Covid_cancer_tf & Covid_cancerless_tf). Training of the models has been performed with the same amount of data (40 M representations/6 M sentences) and with the same training parameters as the original implementation (metapath2vec -train input < input.txt > -output < …/output.txt > -pp 1 size 128 -window 7 -negative -5 -threads 32), leading to similar sized corpus of sentences. Disease vector relations has been calculated with cosine similarity and performance comparison of all results has been made with extended clinical disease-disease comorbidity data to calculate AUROC.

### Disease-disease interaction mapping and ROC score tuning

Jin et al. used a relatively small collection of clinical comorbidity data for validation of mpDisNet, which consisted of a total of 81 unique interactions clinically documented disease comorbidities obtained through a semantic similarity study. We used a manual curation method to find accurate matching between diseases that were titled differently in databases to increase the number of clinically documented comorbid disorders.

Disease names used in the mpDisNet algorithm were gathered from the OMIM database, which includes broad terms for diseases without any specific variations. We aimed to merge this set with data from Ko et al.^15^ which has extended clinical comorbidity data from US-Medicare (PMID:19360091)^16^. The dataset presents over 100 k disease interactions and disease were represented by their ICD-9 codes, hence we systematically matched the names in two sources as follows:

- Disease names retrieved from OMIM database, have only 29 exact matches in Ko et. al. comorbidity list provided in their supplementary material. Exact-match diseases are directly transferred as ICD-9 codes into our dataset.
- For other diseases that could not been matched exactly, disease groups and ICD-9 code ranges of these diseases are extracted from the ICD-9 Data (http://www.icd9data.com/2015/Volume1/default.htm)
- Some of the diseases have been assigned different names in Ko et. al. when compared to the OMIM disease name (i.e.: Esophagitis→Esophageal Reflux). To decrease the number of unmatched diseases, such alternative names were taken into consideration.
- Number of matched diseases are increased from 29 to 170 and when group similarities are also included, we were able to match the 251 of 305 disease names in our modified OMIM list (Supplementary Doc: ICD-9_list.xlsx)
- All these disease interactions have been filtered by Relative Risk > 1.5 criteria which signifying that the patient has more than %50 chance to have the disease-2 if they already have disease-1. At the end, we have collected 4099 unique disease-disease pairs that have a minimum of 1.5 relative risk of occurring together in each patient (Supplementary table: rev_over_15_RR.xlsx).

## Results and discussion

### Network based word-embedding (mpDisNet)

**Figure 1:**
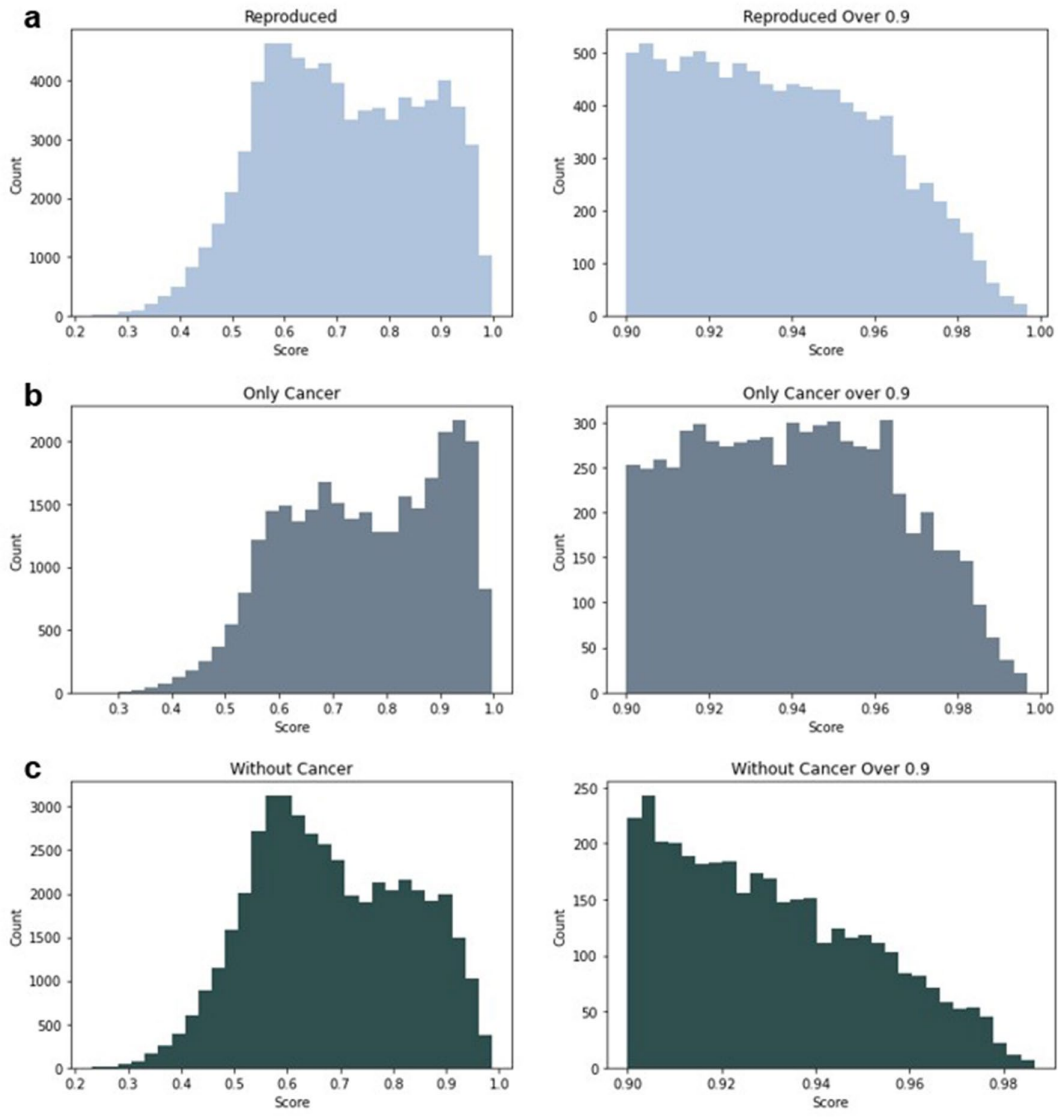
Score distribution of the mpDisNet (reproduction model) that represents the effect of the cancer-term dominance in the disease interaction scores. (**a**) Reproduced model score distribution of all disease scores from the mpDisNet trial. (**b**) Score distributions of cancer-terms in the range between 0.9 and 0.95. (**c**) Score distributions of the remaining (non-cancer) disease interactions.

The OMIM database was used to collect 394 disease types to be used in mpDisNet models. Results from the reproduced model show that, majority of the high-scoring disorders are cancer-related phrases, as can be observed in the reproduction score distribution (Fig. 1). The distribution indicates that, MpDisNet scores are highly biased towards the cancer related terms. (Supplementary doc: Similarity mpdisnet.xlsx), We discovered 10,563 disease-disease associations with a score higher than 0.9, which is computed using vector cosine similarities. 6838 out of 10,593 disease similarities contain at least one cancer related term, which constitutes nearly %65 of the scores higher than 0.9.

Scores of cancer-related phrases, as shown in Fig. 1a, are likewise the main reason for the higher score accumulation between 0.95 and 1. This has a significant impact on the overall distribution of scores for diseases other than cancer. Because of the large amount of cancer-related research and the disease's complications, cancer is strongly linked to all other diseases, resulting in higher comorbidity ratings. Removal of the cancer-related elements from the disease similarity scores reduces the score accumulation on the high score range, as seen in Fig. 1c.

**Figure 2:**
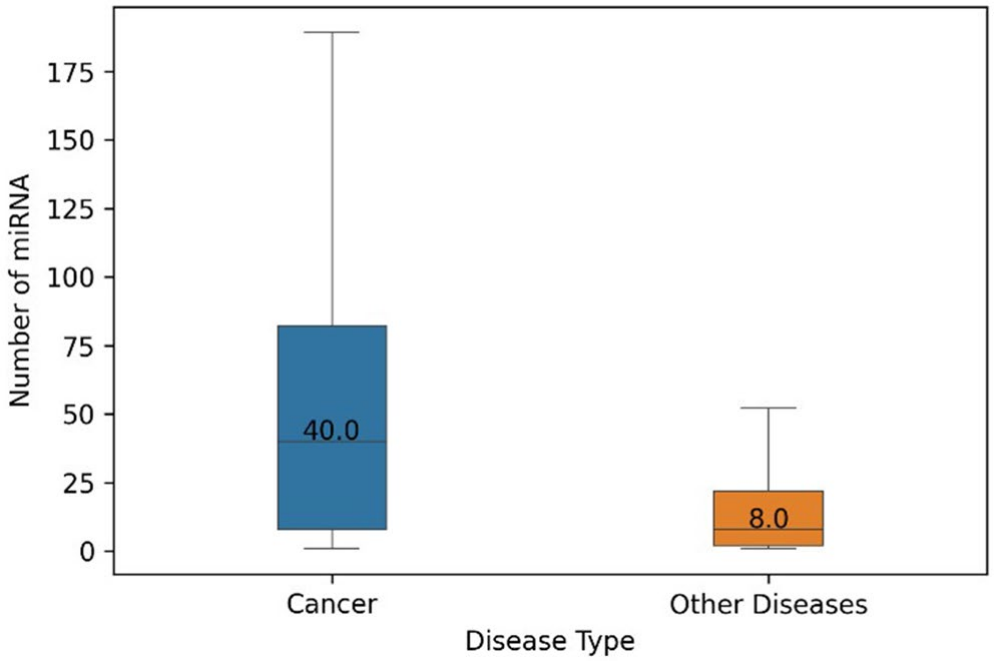
Comparison of the medians of the number of miRNA’s are related with cancer-type diseases and the rest of the OMIM disease dataset.

**Figure 3:**
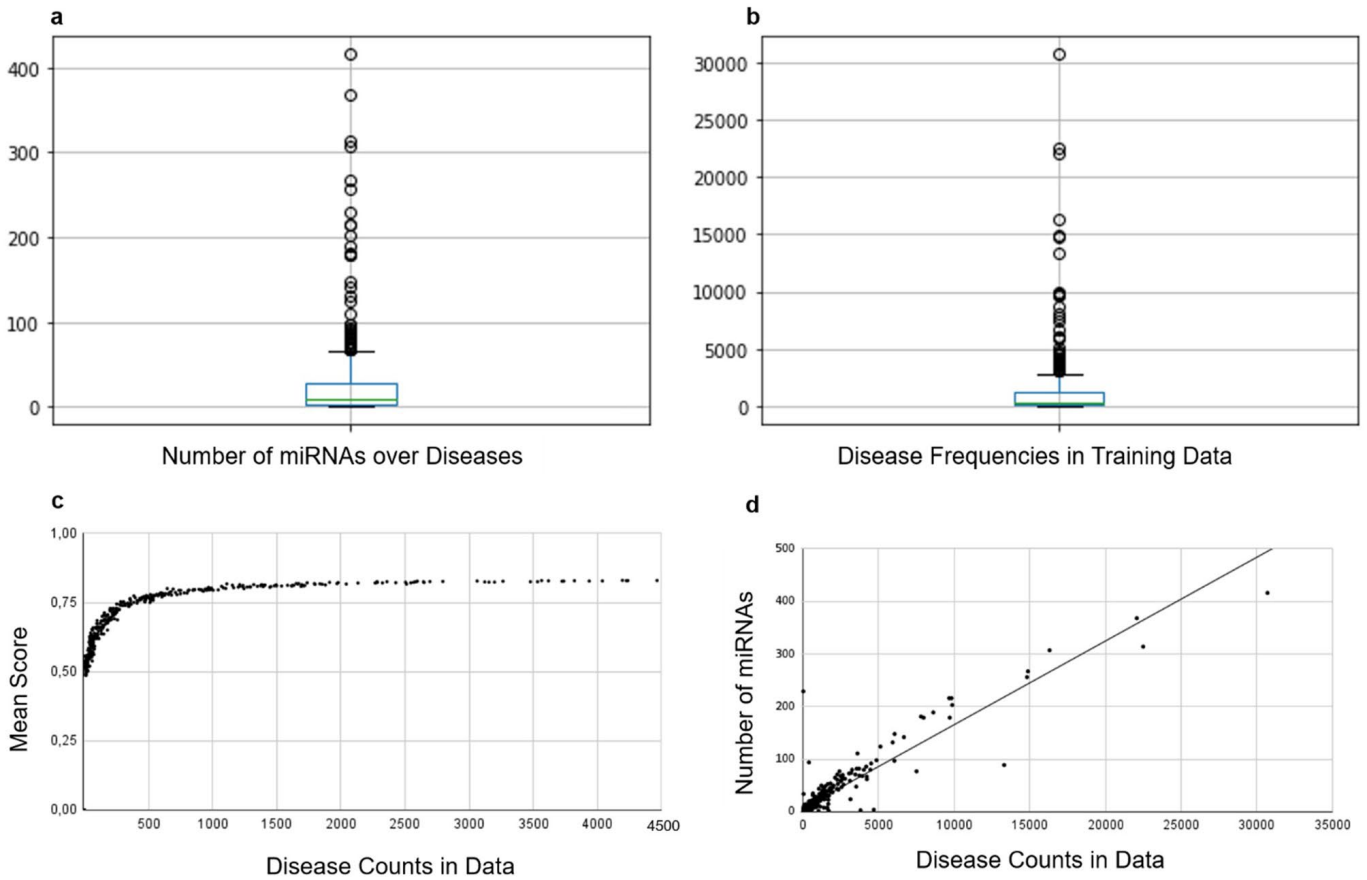
Effects of variations of miRNA counts in diseases and the disease frequencies in the training data. (**a**) Boxplot of the number of the miRNAs of each diseases indicate a narrow IQR range (box) and high number of outliers (circles). (**b**) Occurrence frequencies of each disease in training data in non-modified version. **c** Scatter plot of mean score of each disease and its frequency in the training data. (**d**) Positive correlation between the disease frequencies and number of miRNAs.

When cancer terms and their linked miRNAs are removed from the training data, significant changes in the score distribution is observed. This change in the distribution also indicates that, the number of highly connected elements such as cancer terms also leads to a reduction in the occurrence of other disease representations in the model. Since multiple pathway dysfunctions emerge in cancers, a larger number of related miRNAs were reported in literature. Indeed, as it shown in Table 2, number of discovered miRNAs for cancer terms are large in comparison to median number of miRNAs (Fig. 2) per disease used in this study. Cancer related miRNAs constitute the outlier points in Fig. 3a and lead to high number of occurrences of cancers in training data as shown in Fig. 3b–d. The imbalance in number of miRNAs in cancer and non-cancer diseases lead to dominant occurrence of cancer terms over other diseases, which increases the possibility of random selection of cancers in different sub-networks in the corpus and causes over-training of their vectors.

**Table 2:** Cancer related disease keywords and number of cancer related diseases involved into the training data.

Cancer keyword	Num. of diseases in list	Num. of miRNAs connected
Carcinoma	14	909
Neoplasms	34	2841
Tumor	5	35
Blastoma	5	495
Leukemia	10	464
Lymphoma	11	275
Sarcoma	7	268
Myeloma	1	80
Melanoma	1	229
Glioma	1	219
Total	89	5185

Further, unbalanced occurrence of words (diseases) causes instabilities such as vector update rate disparities between high and low frequency words (Fig. 3). The degree of learning for each condition will eventually be affected by differences in the number of updates of the individual disease vectors^17^. As a result, there will be differences between reliability of disease interaction scores for relatively rare disorders and high frequency disorders. In NLP models, this property can be used to classify the words by their semantic information importance. However, in disease representations, there is no difference between the diseases in terms of information values i.e., diseases cannot be classified as more important or less important in our context as in other NLP problems. This is the main difference between the real words and word-like representations. By removing highly connected diseases, we would like to increase the score reliability of the rest of the diseases and consequently increase the prediction performance.

Prior to applying the approach to the COVID-19 disease to find possible comorbidities, we aimed to increase the disease interaction identification performance. Use of heterogeneous miRNA-gene-disease network approach is mostly conserved in our analysis, which is based on data from miR2Disease and HMDD miRNA-disease interactions^9,18^. The random walk method based on meta-paths has also been preserved. However, changes have been made to increase the accuracy of the network method. In contrast to the original architecture, we used a TF-TF interaction network rather than the PPI to be able to represent the regulatory mechanisms in a more precise manner. The cosine similarity of the disease vectors, which is one of the distance metrics used for quantifying the word similarities in NLP applications, was utilized to analyze disease similarities (comorbidities) for performance evaluation of the method.

### Implementation of COVID miRNA data

miRNA expression profiles of SARS-CoV-2 infected cells have been collected from Wyler dataset^19^. 24-h mock-infected samples were used as control samples. Infected Calu3 cell samples have been analyzed for identification of differentially expressed miRNAs (Calu3 4 h–12 h–24 h). 39 significant and differentially expressed miRNA’s have been identified (adj.p-value < 0.05) which includes hsa-mir-4485, hsa-mir-483 and has-mir-155.

### Updated disease-disease relationship scores

**Figure 4:**
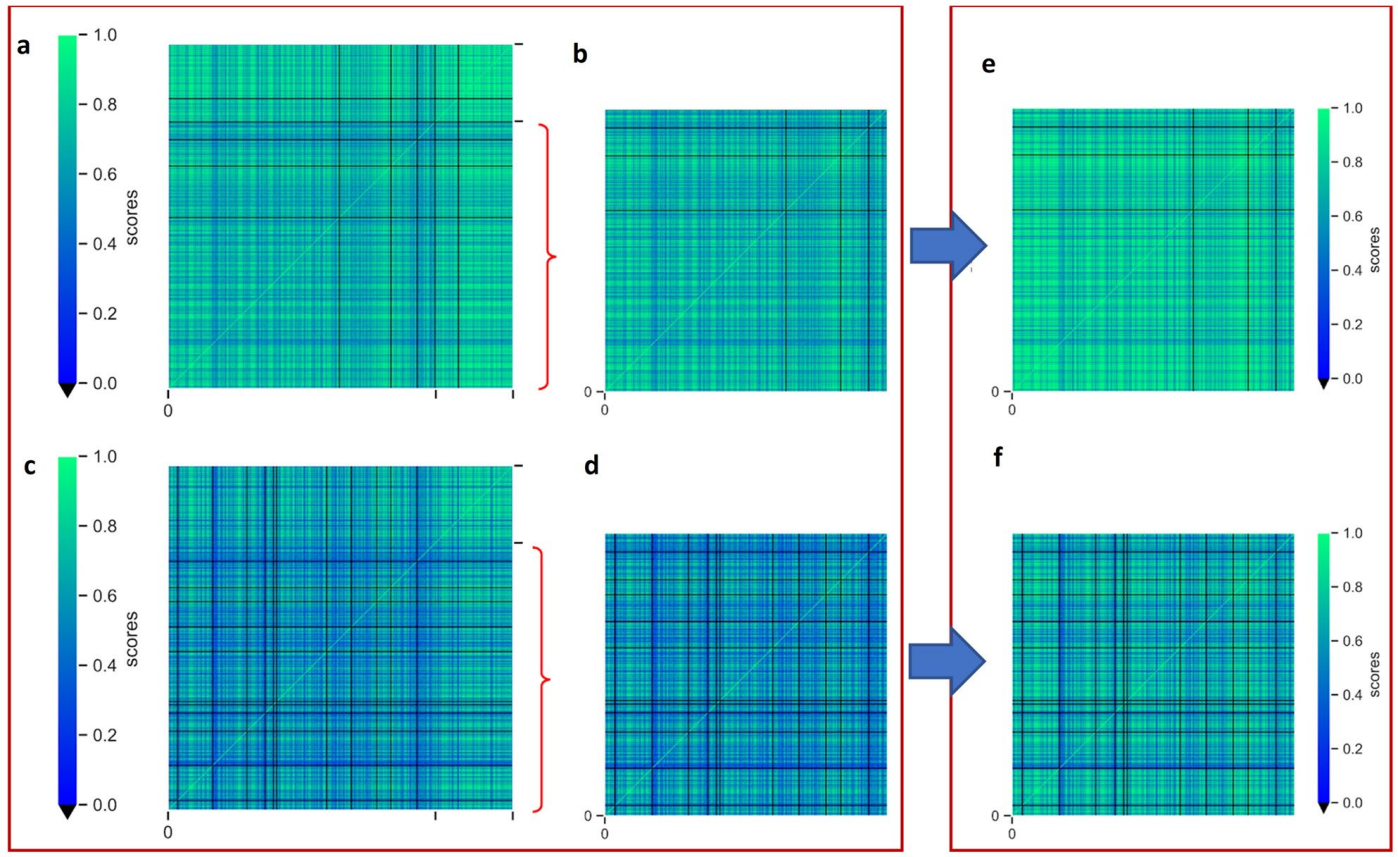
Heatmaps representing the similarity scores in reproduction and TF integrated network. All diseases are placed into x and y axis and they are colored based on their similarity scores as green (1) and blue (0). (**a**) Heatmap of the reproduction scores. (**b**) Scores after cancer-associated diseases are removed (sub-frame of part A that matches with diseases in part E). (**c**) Scores of Transcription-factor integrated model instead of Human PPI with cancer terms. (**d**) TF model without cancer terms (sub-frame of part B that matches with diseases in part F). (**e**) Updated training without cancer terms, with Human PPI, and (**f**) updated training with TF interaction network without cancer terms. Black color represents the zero score that indicates that no association between diseases was found. The region between the black ticks on the x and y axes in (**a**) and (**c**) indicate the cancer (right) and non-cancer (left) diseases.

The score distribution in the reproduced version and in our version with improvements in the disease list and transcription-factor implementation has been shown as a heatmap for all disease scores (Fig. 4).

Excluding cancer terms and their related miRNAs from the heatmap data resulted in higher scores on non-cancer disease relationships. Figure 4a depicts the reproduction score distribution, with cancer-related phrases gathered in the top-right side of the graph, which also has the highest scores. It was transformed into Fig. 4b by deleting cancer-related rows and columns from the heatmap, resulting in a clear distinction between the effect of cancer-related terms on the score distribution. Figure 4e, on the other hand, was produced by retraining disease pair scores after removing cancer diseases and their corresponding miRNA set from the training dataset of the model, it can be seen that the overall score profile for non-cancer diseases has improved. Figure 4c demonstrates the distribution of disease similarity scores including cancer terms when TF-TF interaction data is utilized instead of Human-PPI. In this case, some disease relationships were lost, and the majority of disease scores were reduced. However, scores of the some of the rare disease interactions were increased that may be of significance. Figure 4f demonstrates that when cancer terms are omitted from the TF-TF included trials, the effect on the scores is similar with the upper row, again demonstrating the cancer domination in models where cancer interactions were included. Score distribution differences between the TF-TF regulatory map implementation and PPI can be seen when Fig. 4c and f are compared. Although the scores of the TF network models are lower than the PPI network models, the training time of the models has been greatly decreased due to the smaller vocabulary when TF network is used.

Several diseases were found to have no comorbidity with the rest of the diseases (Table 3). All of these diseases have quite a limited number of miRNA connections in the disease-miRNA data in the network. In the Reproduction and Cancerless models, most of them only have one miRNA interaction. When the TF-TF interaction data was used, the non-comorbid disease list was expanded to include some of two miRNA-connected disorders that are not linked to the transcription factor interactions in the network.

**Table 3:** Zero scored diseases which do not have any connection with any of the other diseases.

Model	Diseases
Reproduction	'Mitochondrial Encephalomyopathies', 'Prostatic Diseases', 'Tauopathies', 'Cystadenocarcinoma, Serous'
Covid_cancerless	'Mitochondrial Encephalomyopathies', 'Prostatic Diseases', 'Tauopathies'
Covid_cancer_tf	'Anodontia', 'Chagas Disease', 'Dyspepsia', 'Frontotemporal Lobar Degeneration', 'Guillain–Barre Syndrome', 'Heart Defects, Congenital', 'Lymphedema', 'Myopia', 'Pemphigus', 'Prostatic Diseases', 'Tauopathies'
Covid_cancerless_tf	'Anodontia', 'Chagas Disease', 'Dyspepsia', 'Frontotemporal Lobar Degeneration', 'Guillain–Barre Syndrome', 'Heart Defects, Congenital', 'Lymphedema', 'Myopia', 'Pemphigus', 'Prostatic Diseases', 'Tauopathies'

### ROC score tuning results

ROC (Receiver Operator Characteristic) curves are often used to evaluate the performances of prediction algorithms by presenting true positive rate and false positive rate of predictions as a curve. For this evaluation, information on true positives and true negatives is needed. Compilation of True Positives (comorbidities) from literature is relatively easy despite the scarcity of verified disease-disease interactions in the literature. However, finding the True Negatives is far more challenging as there is no literature data that directly reports non-comorbid pairs of diseases. One way is designating disease pairs with a low RR score or no interaction information as ‘non-comorbid’. This technique classifies comorbidities not yet reported in the literature as False Positives (FP) in ROC curve calculations. As a result, True-positive (TP) scores are hampered when each disease has a small number of known comorbidities. To address this negative bias on AUROC, more comorbidity data is needed to increase the TP/FP ratio. We expanded the amount of clinical data in the validation set to be used in mpDisNet., as a result, the AUROC performance was greatly improved over the original implementation.

**Figure 5:**
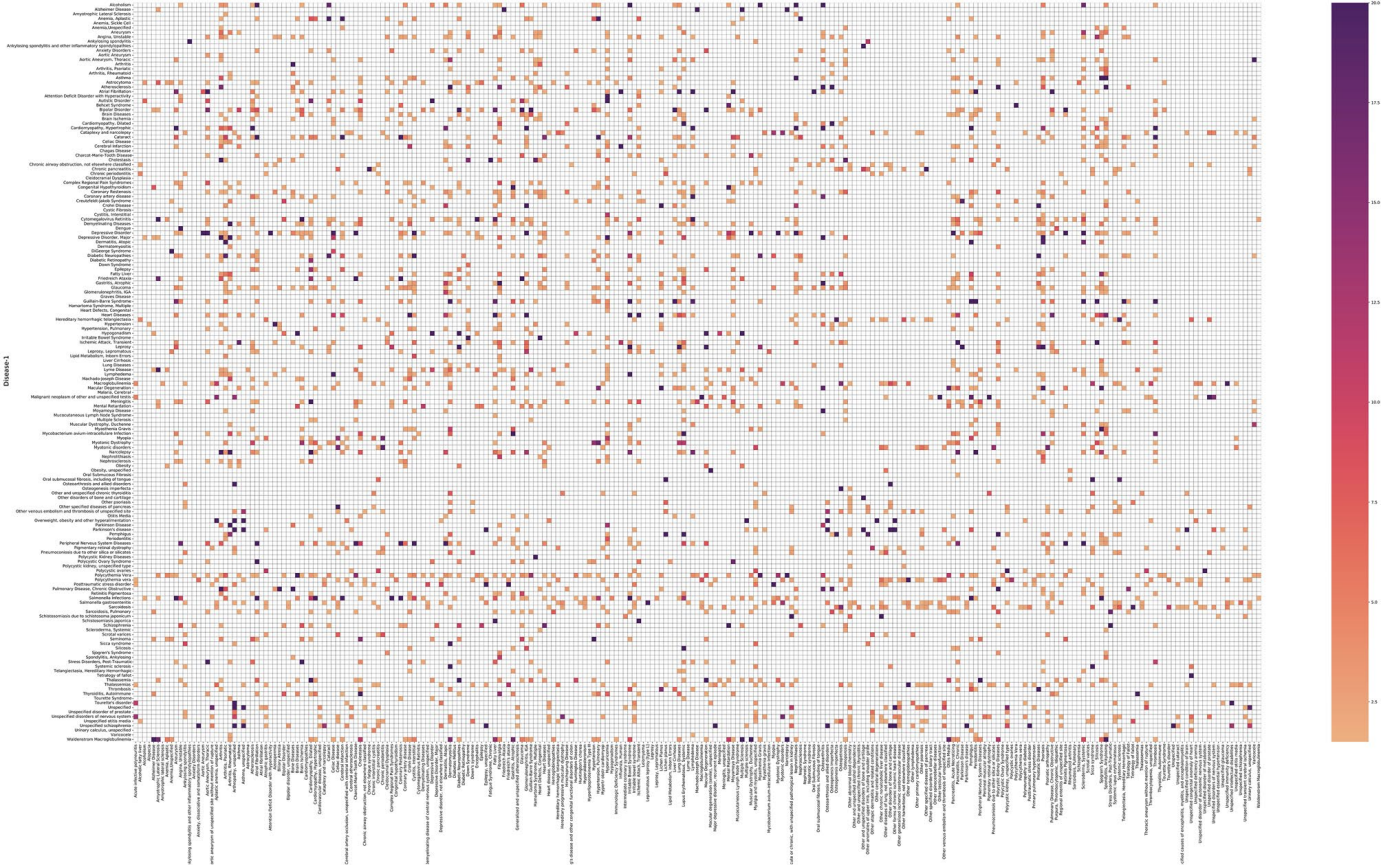
Diseases that have at least 1.5 Relative-Risk (RR) score from US Medicare data visualized as heatmap with matplotlib seaborn python package v3.4. Full sized heatmap can be found in Supplementary Fig. 1 (disease_heatmap.pdf) and full list of disease comorbidity scores from MediCare data can be found in material rev_over_15.xlsx).

**Figure 6:**
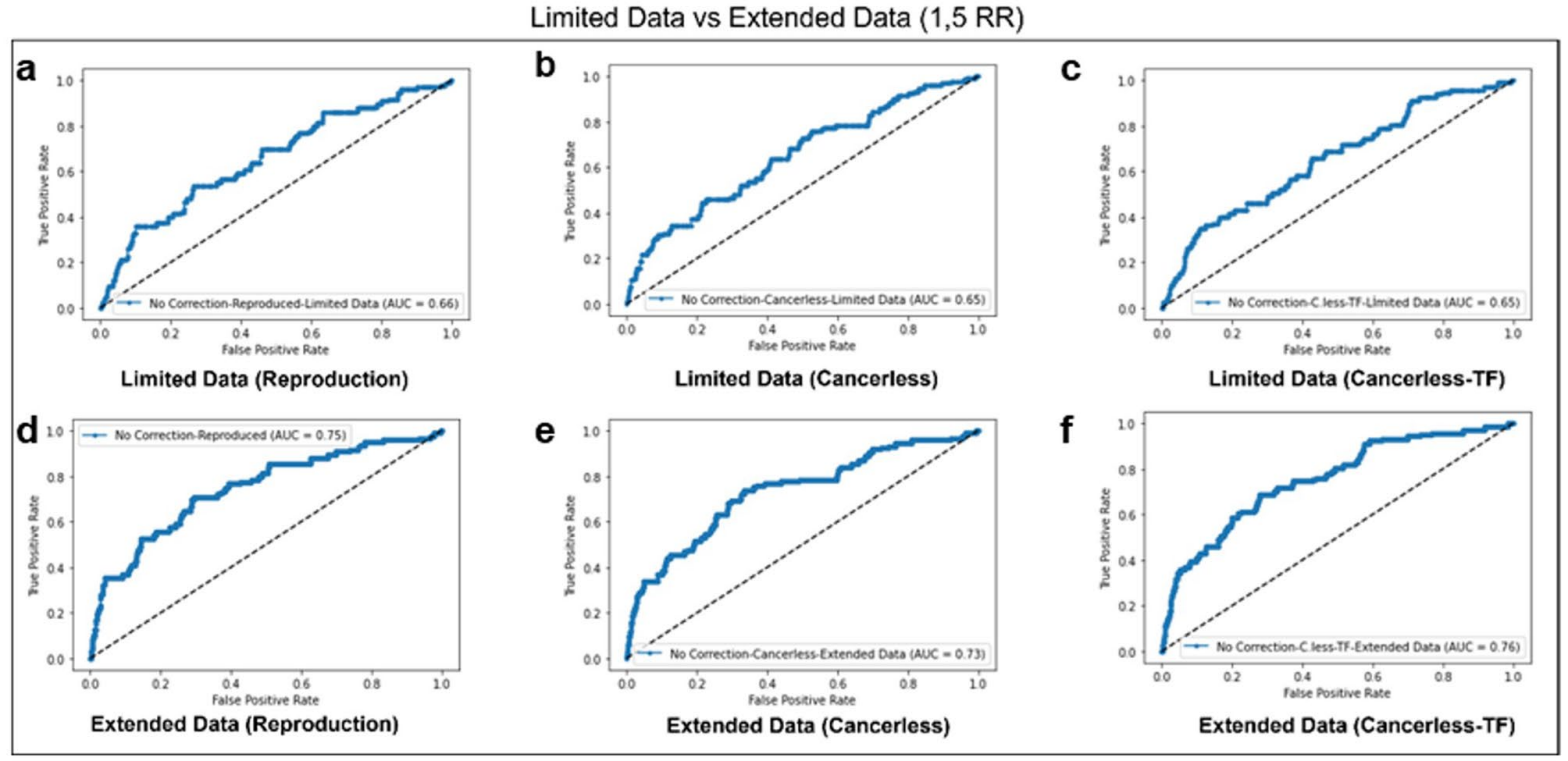
Receiver-Operator Curve (ROC) curve of all models. (**a**) ROC score for reproduced model with same setup in the original MpDisNet implementation. (**b**) ROC curve of cancer removed model scored compared to limited known disease interactions (81 pairs). (**c**) TF-TF substituted model scores compared to limited disease data. (**d**) Reproduction of the original model with extended known disease pairs. (**e**) Cancer removed model with extended disease pairs. (**f**) Cancer removed and TF integrated model with extended disease pairs.

The performance of original implementation of mpDisNet in terms of AUROC (Area Under ROC) was 0.65, which was higher when compared to the AUROC of the overlap method (0.58), a simpler methodology that finds comorbidities by comparing shared miRNA ratios between two disorders, The key drawback of the ROC analysis in the original implementation is the high number of predicted Disease-Disease interactions, which is around 90.000 [n * (n−1)], compared to a small number of known disease interactions which is 81. To be able to expand this list, the disease pairs with RR higher than 1.5 in MediCare dataset and the comorbid disease list of 81 pairs were merged, after all disease names were converted to ICD-9. Generated final Disease-Disease scores (Supplementary file: rev_over_15.xlsx) were converted to pivot table by using pandas python package^20^. The compiled data visualized as heatmap (Fig. 5) with matplotlib v3.4 seaborn python package^21^. Disease similarity scores were used to calculate TP and FP rates when compared to compiled list of comorbidities and ROC curves were drawn for all cases (Fig. 6). The main objective of this improvement is to maximize TPR to better understand the algorithm's true discovery performance. However, since the algorithm's False Discovery Rate cannot be changed, as previously stated, and all disease interactions that have not yet been documented in the literature ought to be labeled as 0, leading to false positives.

To determine whether the implementation of the TF-TF regulation mechanism has a beneficial effect on the discovery of comorbidities, a comparison between the PPI network and the transcription factor-implemented network was made.

Figure 6a presents the reproduction of the original implementation, and the same AUROC is reproduced as expected. The modifications on the model (removal of cancer terms and using TF-TF instead of PPI) did not improve the results as seen in Fig. 6b and c, when they were evaluated using limited clinical data. However, in the second row of Fig. 6, use of extended clinical data significantly improves the AUROC when compared to its counterpart on the first row.

We further hypothesized that correlation between scores of disease pairs may be a more accurate measure of similarity or comorbidity between them. A high positive correlation of scores indicates that the pair of diseases have similar scores with other diseases, hence has a common profile of similarities in their mechanisms. This approach also mitigates the impact of low-frequency disorders having low scores due to lack of miRNA connections.

**Figure 7:**
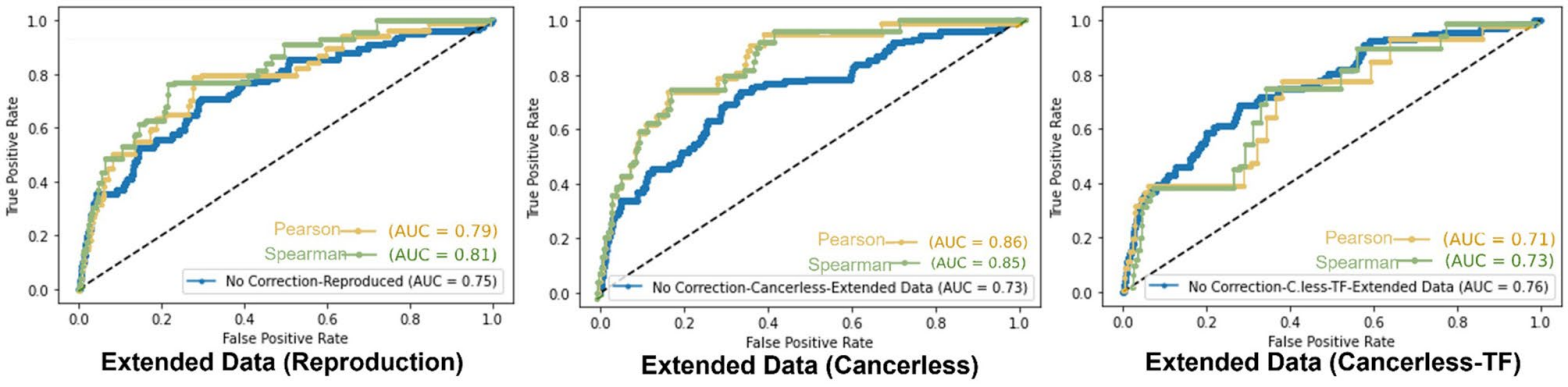
Updated ROC representations of three main approaches (**a**) Reproduction data with No correction (blue), Pearson correlated scores (gold), Spearman correlation (green), (**b**) cancer removed, and correlation implemented. (**c**) Cancer removed and TF integrated network with correlation tunings.

We tested two alternative correlation metrics; Pearson and Spearman correlations are calculated between similarity scores of each disease pair using our mpDisNet results as shown in Fig. 7. Although both metrics produced similar results, to reduce the effect of possible methodological bias, both Pearson and Spearman correlation score-based performances were kept in the ROC curves. When correlations are used for evaluation of performances, we find that cancer-term included model scores also have slightly improved AUROC performance. There is more obvious improvement in Cancerless model than other models. We could say that the similarity between diseases is more prominent when we keep PPI and remove cancer terms as seen in Fig. 7b. In addition, concordance of the Spearman and Person correlations in Cancerless model could be evidence of improved score reliability when compared to the other models. But in order to keep taking into consideration the non-normal distribution of the similarity scores between disease pairs, the non-parametric Spearman correlation coefficients may be more appropriate to keep. Therefore, only Spearman correlation of vector similarity scores were used to determine possible COVID comorbidities in the following section.

### Disease interactions and possible COVID comorbidities

Although our modifications on the algorithm reduced the scores in general, we have observed that low scores of some disease pairs in reproduction model are increased in the modified models. For example, there is an increase in Rheumatoid Arthritis and Depression comorbidity score from 0.79 to 0.90, which is one of the known comorbidities in the literature^22^. Another elevated score is between epilepsy and chronic hepatitis, clinical evidence suggests that these two disorders are strongly comorbid and should be further examined^23^. The algorithm cannot provide any direction information between disease comorbidities, therefore it is not possible to assume causality such as one disease being the cause of another disease, since the direction of the comorbidities cannot be implemented into the network structure yet. However, these findings could indicate that, patients with one disease could have a higher genetic and regulatory inclination to another disease which have high similarity score to the first disease^24^.

We have used our results to investigate comorbidity of COVID-19 with other diseases as a case study. Highly scored diseases that are potentially comorbid with COVID-19 have been retrieved from cancer-removed network training results with Spearman correlation of scores. The threshold has been chosen as 0.9 since it was found as the optimum threshold for the Cancerless model in the ROC curve performance analysis. The algorithm found 156 diseases (Supplementary table: COVID_comorbs.xlsx) with a similarity score of more than 0.9 and correlation of more than 0.95, indicating a strong link to COVID-19 with associated genes and miRNAs. There are also 57 disorders with a score of 0.8 to 0.9, it can be suggested that a moderate link between these diseases and COVID-19 exists. We have identified high-scored associations with disorders that had clinical evidence of increased risk with COVID-19 on the CDC website, such as Diabetes (0.996), Heart Diseases (0.989), Schizophrenia (0.994), and Hypertension (0.994) (https://www.cdc.gov/coronavirus/2019-ncov/science/science-briefs/underlying-evidence-table.html) (Supplementary Dat: CDC_Diseases.xlsx). When the Spearman correlation is applied to the result file, the number of probable COVID disease interactions (Scores over 0.95) increases (From 97 to 210). Also, the overall score of CDC diseases increased from 0.92 (stdev ± 0.054) to 0.98 (stdev ± 0.012).

Encouragingly, we have further found that there are strong links between immune response and infection in diseases such as Hepatitis, Infectious Disorders, and several lung-related diseases. High-scores were also found for vessel and artery-related disorders, such as coronary artery disease, aortic aneurysm, and renal-related diseases. Additionally, various neurological and psychological disorders, such as Alzheimer’s, Parkinson’s, Depression, and Schizophrenia, may raise the impact of COVID-19 according to our results. Indeed, recently this link was shown for Parkinson's Disease in the literature^25^.

### Considerations and limitations in applying NLP approaches to disease similarity modeling

While application of disease similarity networks to the NLP models is a promising approach, there are some challenges that should be tackled. The first of them is biases in data, as stated in the first part of the Results and Discussion, over-representation of diseases such as cancer and subtypes can substantially skew the disease representations as shown in Fig. 1. The choice of network also has an impact on the outcome. Since the final goal is to trace back the disease similarities and identify the potential genes/metabolic activities that mediate the similarities, it is important to keep only the interactions that are mechanistically meaningful. The original reliance on human PPI may not have offered the mechanistic precision that TF-TF interaction network could. While the benefits of integrating TF-TF were not immediately obvious, exploring specific subtypes of regulatory mechanisms in future models could augment performance further. A critical limitation in the initial approach was the scant validation data, confining the model's evaluative robustness. Diseases, influenced by factors like genetics and environment, require a model that captures this complexity. Word2vec and similar embeddings, while powerful, have the risk of oversimplifying these complexities. A holistic view, potentially achievable by assimilating diverse data sources like clinical records and genomic databases, is desirable. While the introduction of correlation metrics illuminates aspects of disease similarity, it is paramount to distinguish between mere correlation and actual causation. Lastly, presented model could provide a quick and broad perspective on disease comorbidities by offering easy implementation. However, while this quick glimpse is valuable in such cases as pandemics, a deeper dive into the underlying causes and intricacies of these disease connections is essential. As we forge ahead, it becomes evident that continuous refinement and validation are not just beneficial but crucial on these applications.

## Conclusion

Vectorization of diseases using a meta-path based on gene regulatory perspective is a simple and promising method for revealing mechanistic similarities between diseases. This strategy has many benefits, such as ease of implementation and availability of a range of meta-path alternatives to increase resolution of mechanistic information represented by the models. A similar approach could be applied to other disease-disease interaction prediction algorithms and the effect of the underlying mechanisms could be identified by expression profiles with integration of relevant meta-paths.

One of the key limitations of such algorithms is the lack of a statistical method that could reliably compare the results. Raw scores can be biased by several factors such as variable representation of each disease in training data, making the comparison even more challenging. However, use of correlation to further relate the disease scores may eliminate some of the bias and provide a significant impact on the general performance. One other drawback of such models is the selection of a gold standard dataset for verification of identified mechanistic similarities. Further, due to scarcity of gene expression profiles from comorbid patients, the validation may rely on only one or two datasets. Design of new studies to collect the data for model validation in comorbid patients could greatly enhance construction and validation of such predictive models of disease comorbidity.

We aimed to overcome some of the mentioned limitations in this work by using alternative meta-paths and evaluation metrics. In addition, increasing the volume of clinical comorbidity data to enhance true positive rates lead to the expected improvement on the algorithm's ROC performance. However, collection and curation of more disease pairs had two major challenges: lack of thorough clinical comorbidity data in the literature and inconsistencies in disease names across databases and literature, which necessitates manual processing of the information. These shortcomings not only make mapping disease-disease linkages more challenging, but they also lead to the conclusion that algorithmically determined disease comorbidities less significant than they are. Nevertheless, by addressing these limitations on performance evaluation criteria and the algorithm's capabilities, newly discovered potential comorbidities of COVID-19 were proposed for novel clinical comorbidity studies.

Detecting potential comorbidities could be a first step toward reducing the number of computational and experimental trials needed in future disease interaction cases. It may also be effective in revealing the mechanistic background of disease interactions. mpDisNet is an innovative and promising strategy for discovering new disease-disease interactions and gaining a better understanding of the mechanical foundation of both novel and also previously known comorbidities. We have addressed some of the limitations in the original algorithm in order to broaden its application to a wider range of scenarios. In a future pandemic, this approach can be rapidly applied to reveal potential comorbidities and allow the national health institutions to prepare for effective care of patients with existing health conditions.

### Supplementary Information

Supplementary Information 1.

Supplementary Figure 1.

Supplementary Information 3.

Supplementary Information 4.

Supplementary Information 5.

Supplementary Information 6.

Supplementary Information 7.

## Data Availability

All data generated or analyzed during this study are included in this published article [and its supplementary information files]. Public RNAseq data were collected from GEO database with the accession number of GSE148729.

## References

[1] Porcheddu R, Serra C, Kelvin D, Kelvin N, Rubino S (2020). Similarity in Case Fatality Rates (CFR) of COVID-19/SARS-COV-2 in Italy and China. J. Infect. Dev. Ctries..

[2] Sadegh S (2023). Lacking mechanistic disease definitions and corresponding association data hamper progress in network medicine and beyond. Nat. Commun..

[3] Yang J (2020). Molecular interaction and inhibition of SARS-CoV-2 binding to the ACE2 receptor. Nat. Commun..

[4] Spataro N, Rodríguez JA, Navarro A, Bosch E (2017). Properties of human disease genes and the role of genes linked to Mendelian disorders in complex disease aetiology. Hum. Mol. Genet..

[5] Capobianco E, Lio P (2013). Comorbidity: A multidimensional approach. Trends Mol. Med..

[6] Keshava Prasad TS (2009). Human protein reference database—2009 update. Nucleic Acids Res..

[7] Lee DS, Park J, Kay KA, Christakis NA, Oltvai ZN, Barabási AL (2008). The implications of human metabolic network topology for disease comorbidity. Proc. Natl. Acad. Sci..

[8] Jin S (2019). A network-based approach to uncover microRNA-mediated disease comorbidities and potential pathobiological implications. npj Syst. Biol. Appl..

[9] Jiang Q (2009). miR2Disease: A manually curated database for microRNA deregulation in human disease. Nucleic Acids Res..

[10] Hamosh A, Scott AF, Amberger JS, Bocchini CA, McKusick VA (2005). Online Mendelian Inheritance in Man (OMIM), a knowledgebase of human genes and genetic disorders. Nucleic Acids Res.

[11] Hermjakob H (2004). IntAct: An open source molecular interaction database. Nucleic Acids Res..

[12] Cowley MJ (2012). PINA v2.0: Mining interactome modules. Nucleic Acids Res..

[13] Marbach D, Lamparter D, Quon G, Kellis M, Kutalik Z, Bergmann S (2016). Tissue-specific regulatory circuits reveal variable modular perturbations across complex diseases. Nat Methods.

[14] Rouillard AD (2016). The harmonizome: A collection of processed datasets gathered to serve and mine knowledge about genes and proteins. Database.

[15] Ko Y, Cho M, Lee J-S, Kim J (2016). Identification of disease comorbidity through hidden molecular mechanisms. Sci. Rep..

[16] Hidalgo CA, Blumm N, Barabási AL, Christakis NA (2009). A dynamic network approach for the study of human phenotypes. PLoS Comput. Biol..

[17] Li B, Drozd A, Guo Y, Liu T, Matsuoka S, Du X (2019). Scaling Word2Vec on big corpus. Data Sci. Eng..

[18] Li Y (2014). HMDD v2.0: A database for experimentally supported human microRNA and disease associations. Nucleic Acids Res..

[19] Wyler E (2021). Transcriptomic profiling of SARS-CoV-2 infected human cell lines identifies HSP90 as target for COVID-19 therapy. iScience.

[20] Mckinney, W. Data Structures for Statistical Computing in Python (2010)..

[21] Waskom ML (2021). seaborn: Statistical data visualization. J. Open Source Softw..

[22] Margaretten M, Julian L, Katz P, Yelin E (2011). Depression in patients with rheumatoid arthritis: Description, causes and mechanisms. Int. J. Clin. Rheumtol..

[23] Chiu HY, Hsieh CF, Chiang YT, Huang WF, Tsai TF (2016). The risk of chronic pancreatitis in patients with psoriasis: A population-based cohort study. PLoS ONE.

[24] Baerwald C, Manger B, Hueber A (2019). Depression as comorbidity of rheumatoid arthritis. Z. Rheumatol..

[25] Semerdzhiev SA, Fakhree MAA, Segers-Nolten I, Blum C, Claessens MMAE (2022). Interactions between SARS-CoV-2 N-protein and α-synuclein accelerate amyloid formation. ACS Chem. Neurosci..

