# Supplementary material for: Optimized network based natural language processing approach to reveal disease comorbidities in COVID-19
Source: Sci Rep. 2024 Jan 28;14:2325. doi: 10.1038/s41598-024-52819-5 (PMC10822845; doi:10.1038/s41598-024-52819-5)
Supplement: Supplementary file 2 — Supplementary Figure 1. [file 41598_2024_52819_MOESM2_ESM.pdf]

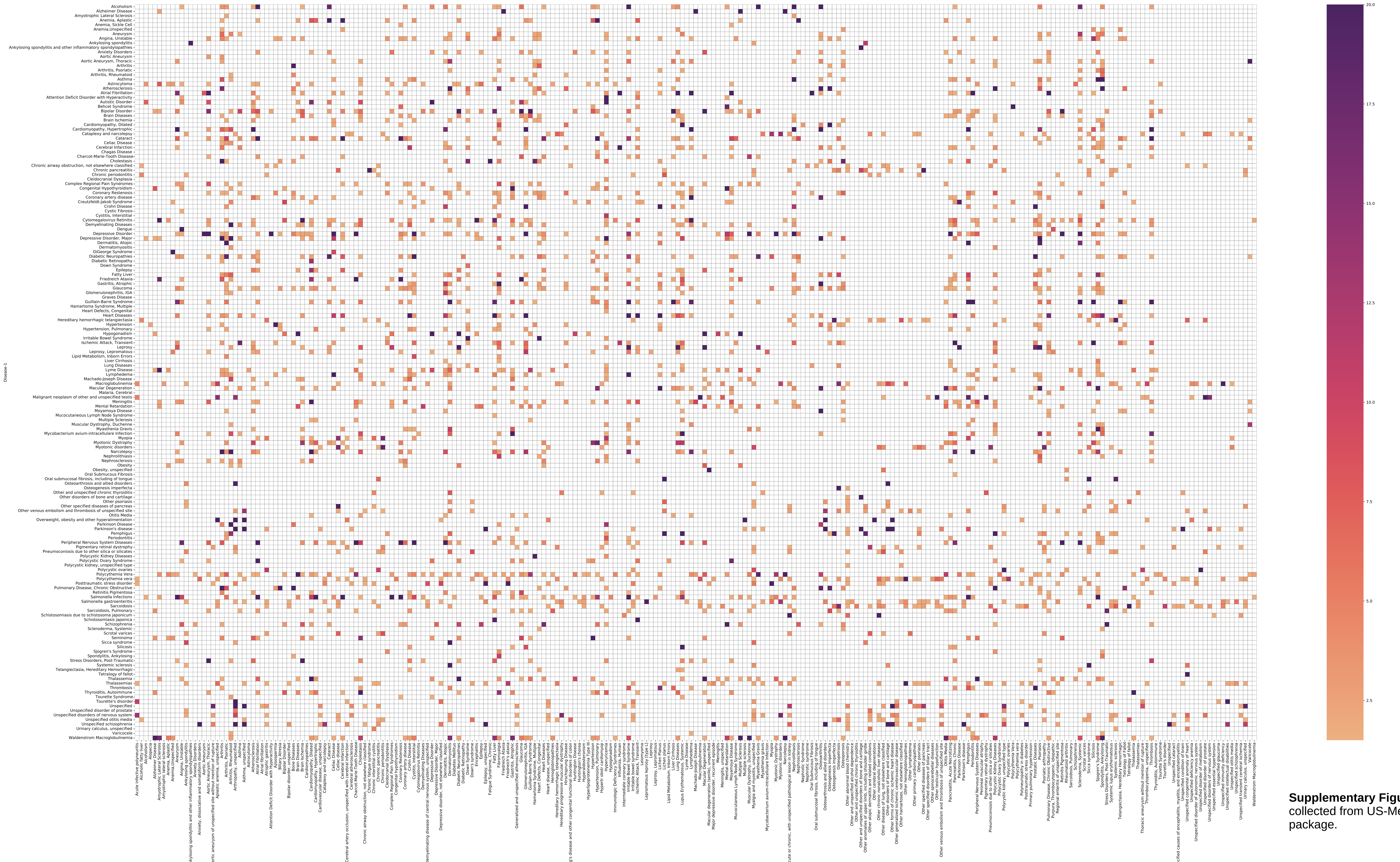

**Supplementary Figure-1:** Heatmap of Disease-Disease interaction scores collected from US-Medicare Dataset and visualized by python seaborn package.
