# Supplementary material for: Optimized network based natural language processing approach to reveal disease comorbidities in COVID-19
Source: Sci Rep. 2024 Jan 28;14:2325. doi: 10.1038/s41598-024-52819-5 (PMC10822845; doi:10.1038/s41598-024-52819-5)
Supplement: Supplementary file 4 — Supplementary Information 4. [file 41598_2024_52819_MOESM4_ESM.pdf]

miRNAs	baseMean	log2FoldChange	lfcSE	stat	pvalue	padj
hsa-miR-6891-5p	35.01686974	1.424242771	1.027678731	108.7414956	2.05E-23	1.75E-21
hsa-miR-483-3p	31.49237991	1.363893906	1.663903967	147.4024307	9.57E-32	1.64E-29
hsa-miR-4449	87.33878319	1.289179343	0.343179418	15.03537017	0.001786671	0.018516407
hsa-miR-23a-5p	93.26957596	1.105208514	0.353046313	13.49921942	0.003672472	0.03394555
hsa-let-7i-3p	184.1354548	1.094165934	0.286040885	14.50789083	0.002289352	0.022370242
hsa-miR-107	125.858376	0.98196456	0.363705109	16.28756581	0.000989972	0.010921631
hsa-miR-103a-3p	905.6557897	0.903197897	0.257494448	22.40995992	5.36E-05	0.000872829
hsa-let-7i-5p	4273.728274	0.88625592	0.206287376	23.0177402	4.00E-05	0.000684695
hsa-miR-181a-5p	1522.500011	0.836577134	0.231391818	15.21730989	0.001640052	0.017528052
hsa-miR-17-5p	399.7199353	0.564228459	0.253483262	19.59983035	0.00020544	0.002810419
hsa-miR-93-5p	1131.679992	0.534610732	0.240407885	13.15278517	0.004317583	0.038858245
hsa-miR-12136	111.6352991	-0.077821174	0.342843992	46.49614222	4.45E-10	1.90E-08
hsa-miR-4284	67.64688929	-0.306926278	0.447922282	86.84987699	1.04E-18	7.11E-17
hsa-miR-155-5p	32.72558406	-0.390960668	0.553900833	19.44060055	0.00022164	0.00291542
hsa-miR-23b-3p	9368.699706	-0.532635212	0.232377653	20.10674964	0.000161311	0.00239863
hsa-miR-155-3p	114.6993601	-0.552412982	0.529999567	118.2443999	1.84E-25	2.10E-23
hsa-miR-29b-3p	1629.800084	-0.574123946	0.198964065	12.89514024	0.004868946	0.042696914
hsa-miR-4463	12.74372968	-0.635921022	1.612923431	51.0929971	4.67E-11	2.28E-09
hsa-miR-25-3p	4481.816302	-0.682679685	0.184620859	14.58018187	0.002212941	0.022259579
hsa-miR-15b-5p	919.1550894	-0.824423749	0.210681054	24.95982144	1.57E-05	0.000336487
hsa-miR-4485-3p	734.646703	-0.824689504	0.259347995	592.8909907	3.50E-128	1.20E-125
hsa-miR-625-3p	352.8107949	-0.911629534	0.272558929	14.17659253	0.002674373	0.025406546
hsa-miR-200b-3p	1941.809136	-0.933107941	0.246004767	16.32407587	0.000973039	0.010921631
hsa-miR-15b-3p	642.4271287	-1.030374054	0.232242709	25.21088262	1.39E-05	0.000318051
hsa-miR-10a-5p	1528.4681	-1.03581559	0.284995115	17.18690444	0.000646862	0.007628506
hsa-miR-7-1-3p	238.6297663	-1.196809759	0.307501349	23.64786613	2.96E-05	0.000562136
hsa-miR-151a-3p	3942.186095	-1.249936529	0.300576234	20.17904892	0.000155839	0.00239863
hsa-miR-16-2-3p	335.7056147	-1.258558871	0.234419867	38.88657794	1.83E-08	5.70E-07
hsa-miR-194-5p	443.8396319	-1.277380247	0.232203665	42.68948543	2.86E-09	1.09E-07
hsa-miR-365a-3p	400.1502899	-1.286292725	0.331746556	19.19643367	0.000248984	0.003041163
hsa-miR-365b-3p	400.1502899	-1.286292725	0.331746556	19.19643367	0.000248984	0.003041163
hsa-miR-135b-5p	228.3181397	-1.399866293	0.309534018	28.69428665	2.60E-06	6.34E-05
hsa-miR-5701	157.9278174	-1.40275953	0.309141107	24.10684529	2.37E-05	0.000477376
hsa-miR-429	1784.936267	-1.410518057	0.293586061	29.03360435	2.20E-06	5.80E-05
hsa-miR-21-5p	15623.5877	-1.424234116	0.284424376	31.02297995	8.41E-07	2.40E-05
hsa-let-7a-3p	1020.649209	-1.429243851	0.327087139	39.00654635	1.73E-08	5.70E-07
hsa-miR-374a-3p	45.7491785	-1.918759558	0.508378473	23.41910643	3.30E-05	0.000594407
hsa-miR-4454	19.20841149	-2.488158307	0.683042865	19.66887363	0.000198787	0.002810419
hsa-miR-374a-5p	180.3824349	-2.874232464	0.397388388	69.0954344	6.67E-15	3.80E-13
