# Supplementary material for: Optimized network based natural language processing approach to reveal disease comorbidities in COVID-19
Source: Sci Rep. 2024 Jan 28;14:2325. doi: 10.1038/s41598-024-52819-5 (PMC10822845; doi:10.1038/s41598-024-52819-5)
Supplement: Supplementary file 6 — Supplementary Information 6. [file 41598_2024_52819_MOESM6_ESM.pdf]

disease1	disease2	similarity	disease1_name	disease2_name
58	229	0,997533788	Carcinoma, Hepatocellular	Lung Neoplasms
58	250	0,997527643	Carcinoma, Hepatocellular	Melanoma
5	149	0,997523038	Adrenocortical Carcinoma	Glioma
62	149	0,997522959	Carcinoma, Renal Cell	Glioma
58	213	0,997517068	Carcinoma, Hepatocellular	Leukemia, Myelocytic, Acute
110	149	0,997472094	Diabetes Mellitus, Type 2	Glioma
58	386	0,997452398	Carcinoma, Hepatocellular	Uterine Cervical Neoplasms
149	213	0,997436955	Glioma	Leukemia, Myelocytic, Acute
149	298	0,997421926	Glioma	Osteosarcoma
149	229	0,99739112	Glioma	Lung Neoplasms
58	149	0,997375836	Carcinoma, Hepatocellular	Glioma
58	67	0,997336356	Carcinoma, Hepatocellular	Cardiomyopathy, Hypertrophic
167	213	0,997331836	Hepatitis B	Leukemia, Myelocytic, Acute
209	213	0,997320642	Leukemia, B-Cell, Chronic	Leukemia, Myelocytic, Acute
110	250	0,997252206	Diabetes Mellitus, Type 2	Melanoma
213	365	0,997250842	Leukemia, Myelocytic, Acute	Stomach Neoplasms
149	386	0,9972383	Glioma	Uterine Cervical Neoplasms
365	386	0,99722442	Stomach Neoplasms	Uterine Cervical Neoplasms
8	213	0,997207342	Alzheimer Disease	Leukemia, Myelocytic, Acute
213	386	0,997182687	Leukemia, Myelocytic, Acute	Uterine Cervical Neoplasms
249	250	0,997181317	Medulloblastoma	Melanoma
149	250	0,997175723	Glioma	Melanoma
5	110	0,997164248	Adrenocortical Carcinoma	Diabetes Mellitus, Type 2
8	58	0,997149148	Alzheimer Disease	Carcinoma, Hepatocellular
149	365	0,997138913	Glioma	Stomach Neoplasms
323	386	0,997121203	Pre-Eclampsia	Uterine Cervical Neoplasms
5	386	0,997119417	Adrenocortical Carcinoma	Uterine Cervical Neoplasms
250	365	0,997105422	Melanoma	Stomach Neoplasms
58	365	0,997101852	Carcinoma, Hepatocellular	Stomach Neoplasms
110	168	0,997083559	Diabetes Mellitus, Type 2	Hepatitis C
5	250	0,997073667	Adrenocortical Carcinoma	Melanoma
5	58	0,997072557	Adrenocortical Carcinoma	Carcinoma, Hepatocellular
5	298	0,997066997	Adrenocortical Carcinoma	Osteosarcoma
215	250	0,997060494	Leukemia, Myeloid, Chronic	Melanoma
298	365	0,99704	Osteosarcoma	Stomach Neoplasms
213	250	0,997018667	Leukemia, Myelocytic, Acute	Melanoma
213	229	0,997012851	Leukemia, Myelocytic, Acute	Lung Neoplasms
250	298	0,997004096	Melanoma	Osteosarcoma
250	386	0,997003703	Melanoma	Uterine Cervical Neoplasms
62	298	0,997000356	Carcinoma, Renal Cell	Osteosarcoma
229	282	0,996989034	Lung Neoplasms	Neuroblastoma
61	386	0,996975282	Carcinoma, Pancreatic Ductal	Uterine Cervical Neoplasms
8	250	0,996974753	Alzheimer Disease	Melanoma
298	386	0,996970177	Osteosarcoma	Uterine Cervical Neoplasms
250	282	0,996959683	Melanoma	Neuroblastoma
110	229	0,996954195	Diabetes Mellitus, Type 2	Lung Neoplasms
129	149	0,996954046	Esophageal Neoplasms	Glioma
58	298	0,996945828	Carcinoma, Hepatocellular	Osteosarcoma
229	365	0,996942916	Lung Neoplasms	Stomach Neoplasms
168	282	0,996934143	Hepatitis C	Neuroblastoma
213	235	0,996925591	Leukemia, Myelocytic, Acute	Lymphoma
61	250	0,996895026	Carcinoma, Pancreatic Ductal	Melanoma
168	229	0,996891774	Hepatitis C	Lung Neoplasms
5	213	0,996881785	Adrenocortical Carcinoma	Leukemia, Myelocytic, Acute
8	229	0,996879695	Alzheimer Disease	Lung Neoplasms
129	229	0,996868646	Esophageal Neoplasms	Lung Neoplasms
213	298	0,99686714	Leukemia, Myelocytic, Acute	Osteosarcoma
67	213	0,996864446	Cardiomyopathy, Hypertrophic	Leukemia, Myelocytic, Acute
110	213	0,996854071	Diabetes Mellitus, Type 2	Leukemia, Myelocytic, Acute
61	323	0,99685015	Carcinoma, Pancreatic Ductal	Pre-Eclampsia
61	167	0,996848052	Carcinoma, Pancreatic Ductal	Hepatitis B
51	58	0,99684671	Breast Neoplasms	Carcinoma, Hepatocellular
149	209	0,99684447	Glioma	Leukemia, B-Cell, Chronic
58	323	0,996841736	Carcinoma, Hepatocellular	Pre-Eclampsia
58	167	0,996831455	Carcinoma, Hepatocellular	Hepatitis B
58	61	0,996828731	Carcinoma, Hepatocellular	Carcinoma, Pancreatic Ductal
213	385	0,996826158	Leukemia, Myelocytic, Acute	Urinary Bladder Neoplasms
235	298	0,996816828	Lymphoma	Osteosarcoma

110	211	0,996809116	Diabetes Mellitus, Type 2	Leukemia, Lymphocytic, Acute
67	250	0,996806813	Cardiomyopathy, Hypertrophic	Melanoma
67	149	0,996797656	Cardiomyopathy, Hypertrophic	Glioma
149	282	0,996794415	Glioma	Neuroblastoma
62	365	0,996785427	Carcinoma, Renal Cell	Stomach Neoplasms
168	250	0,996779685	Hepatitis C	Melanoma
67	209	0,9967774	Cardiomyopathy, Hypertrophic	Leukemia, B-Cell, Chronic
58	110	0,996776868	Carcinoma, Hepatocellular	Diabetes Mellitus, Type 2
249	365	0,996776163	Medulloblastoma	Stomach Neoplasms
51	365	0,996767787	Breast Neoplasms	Stomach Neoplasms
149	329	0,996765328	Glioma	Prostatic Neoplasms
61	229	0,996760204	Carcinoma, Pancreatic Ductal	Lung Neoplasms
229	250	0,996750742	Lung Neoplasms	Melanoma
5	91	0,996746927	Adrenocortical Carcinoma	Coronary artery disease
5	365	0,99673356	Adrenocortical Carcinoma	Stomach Neoplasms
110	282	0,996729041	Diabetes Mellitus, Type 2	Neuroblastoma
8	167	0,996727868	Alzheimer Disease	Hepatitis B
62	213	0,996718146	Carcinoma, Renal Cell	Leukemia, Myelocytic, Acute
61	213	0,996711745	Carcinoma, Pancreatic Ductal	Leukemia, Myelocytic, Acute
58	62	0,996710742	Carcinoma, Hepatocellular	Carcinoma, Renal Cell
8	282	0,996708093	Alzheimer Disease	Neuroblastoma
110	129	0,99670662	Diabetes Mellitus, Type 2	Esophageal Neoplasms
62	386	0,996704582	Carcinoma, Renal Cell	Uterine Cervical Neoplasms
110	386	0,996698885	Diabetes Mellitus, Type 2	Uterine Cervical Neoplasms
213	329	0,996698883	Leukemia, Myelocytic, Acute	Prostatic Neoplasms
8	62	0,996687095	Alzheimer Disease	Carcinoma, Renal Cell
292	323	0,996683902	Osteoarthritis	Pre-Eclampsia
51	229	0,996675092	Breast Neoplasms	Lung Neoplasms
149	323	0,996671968	Glioma	Pre-Eclampsia
58	271	0,996664422	Carcinoma, Hepatocellular	Myocardial Infarction
110	365	0,9966552	Diabetes Mellitus, Type 2	Stomach Neoplasms
67	229	0,996654289	Cardiomyopathy, Hypertrophic	Lung Neoplasms
168	213	0,996641447	Hepatitis C	Leukemia, Myelocytic, Acute
213	292	0,996638299	Leukemia, Myelocytic, Acute	Osteoarthritis
209	365	0,996630918	Leukemia, B-Cell, Chronic	Stomach Neoplasms
229	298	0,996626104	Lung Neoplasms	Osteosarcoma
110	298	0,996618733	Diabetes Mellitus, Type 2	Osteosarcoma
58	168	0,996612438	Carcinoma, Hepatocellular	Hepatitis C
61	110	0,996591807	Carcinoma, Pancreatic Ductal	Diabetes Mellitus, Type 2
86	250	0,996591306	Colorectal Neoplasms	Melanoma
58	230	0,996588051	Carcinoma, Hepatocellular	Lupus Erythematosus, Systemic
58	209	0,996585959	Carcinoma, Hepatocellular	Leukemia, B-Cell, Chronic
91	365	0,996581488	Coronary artery disease	Stomach Neoplasms
110	209	0,996578527	Diabetes Mellitus, Type 2	Leukemia, B-Cell, Chronic
5	282	0,996563262	Adrenocortical Carcinoma	Neuroblastoma
365	385	0,996561876	Stomach Neoplasms	Urinary Bladder Neoplasms
250	350	0,996560268	Melanoma	Sarcoma, Kaposi
5	302	0,996554477	Adrenocortical Carcinoma	Ovarian Neoplasms
61	149	0,996551506	Carcinoma, Pancreatic Ductal	Glioma
58	215	0,996545421	Carcinoma, Hepatocellular	Leukemia, Myeloid, Chronic
213	249	0,996538265	Leukemia, Myelocytic, Acute	Medulloblastoma
8	149	0,996538163	Alzheimer Disease	Glioma
91	298	0,996533006	Coronary artery disease	Osteosarcoma
288	365	0,996530646	Obesity	Stomach Neoplasms
213	215	0,996527569	Leukemia, Myelocytic, Acute	Leukemia, Myeloid, Chronic
5	168	0,996527247	Adrenocortical Carcinoma	Hepatitis C
58	282	0,996522663	Carcinoma, Hepatocellular	Neuroblastoma
5	61	0,996516717	Adrenocortical Carcinoma	Carcinoma, Pancreatic Ductal
149	302	0,99651254	Glioma	Ovarian Neoplasms
58	292	0,996511701	Carcinoma, Hepatocellular	Osteoarthritis
58	249	0,996511382	Carcinoma, Hepatocellular	Medulloblastoma
58	330	0,99650692	Carcinoma, Hepatocellular	Psoriasis
167	250	0,996501548	Hepatitis B	Melanoma
215	282	0,996500123	Leukemia, Myeloid, Chronic	Neuroblastoma
5	330	0,996491074	Adrenocortical Carcinoma	Psoriasis
5	67	0,996488582	Adrenocortical Carcinoma	Cardiomyopathy, Hypertrophic
110	271	0,996476897	Diabetes Mellitus, Type 2	Myocardial Infarction
61	365	0,996473053	Carcinoma, Pancreatic Ductal	Stomach Neoplasms
230	282	0,996472583	Lupus Erythematosus, Systemic	Neuroblastoma

124	250	0,996465184	Endometriosis	Melanoma
129	213	0,996456768	Esophageal Neoplasms	Leukemia, Myelocytic, Acute
149	249	0,996453232	Glioma	Medulloblastoma
8	298	0,996452059	Alzheimer Disease	Osteosarcoma
61	168	0,996444556	Carcinoma, Pancreatic Ductal	Hepatitis C
249	282	0,996443404	Medulloblastoma	Neuroblastoma
110	249	0,996441304	Diabetes Mellitus, Type 2	Medulloblastoma
58	91	0,996440741	Carcinoma, Hepatocellular	Coronary artery disease
149	168	0,996438455	Glioma	Hepatitis C
235	250	0,99643697	Lymphoma	Melanoma
58	350	0,996424764	Carcinoma, Hepatocellular	Sarcoma, Kaposi
62	110	0,996421993	Carcinoma, Renal Cell	Diabetes Mellitus, Type 2
168	298	0,996416281	Hepatitis C	Osteosarcoma
110	215	0,996414965	Diabetes Mellitus, Type 2	Leukemia, Myeloid, Chronic
84	282	0,99641492	Colitis, Ulcerative	Neuroblastoma
8	61	0,996414244	Alzheimer Disease	Carcinoma, Pancreatic Ductal
51	213	0,99640823	Breast Neoplasms	Leukemia, Myelocytic, Acute
5	62	0,996399961	Adrenocortical Carcinoma	Carcinoma, Renal Cell
230	298	0,996398934	Lupus Erythematosus, Systemic	Osteosarcoma
149	235	0,996398792	Glioma	Lymphoma
128	149	0,996396634	Epilepsy	Glioma
235	386	0,99639604	Lymphoma	Uterine Cervical Neoplasms
213	330	0,99639541	Leukemia, Myelocytic, Acute	Psoriasis
58	108	0,996394381	Carcinoma, Hepatocellular	Diabetes Mellitus
149	211	0,996391747	Glioma	Leukemia, Lymphocytic, Acute
329	365	0,996389185	Prostatic Neoplasms	Stomach Neoplasms
167	229	0,996387578	Hepatitis B	Lung Neoplasms
249	298	0,99638636	Medulloblastoma	Osteosarcoma
8	365	0,996385459	Alzheimer Disease	Stomach Neoplasms
8	386	0,996384583	Alzheimer Disease	Uterine Cervical Neoplasms
213	323	0,996382207	Leukemia, Myelocytic, Acute	Pre-Eclampsia
323	365	0,996380925	Pre-Eclampsia	Stomach Neoplasms
110	350	0,99637368	Diabetes Mellitus, Type 2	Sarcoma, Kaposi
215	249	0,996372385	Leukemia, Myeloid, Chronic	Medulloblastoma
282	298	0,996370604	Neuroblastoma	Osteosarcoma
8	209	0,996367925	Alzheimer Disease	Leukemia, B-Cell, Chronic
8	67	0,996367778	Alzheimer Disease	Cardiomyopathy, Hypertrophic
5	288	0,996367395	Adrenocortical Carcinoma	Obesity
288	298	0,996364106	Obesity	Osteosarcoma
209	323	0,996362639	Leukemia, B-Cell, Chronic	Pre-Eclampsia
5	229	0,996359664	Adrenocortical Carcinoma	Lung Neoplasms
211	250	0,99635962	Leukemia, Lymphocytic, Acute	Melanoma
8	323	0,996357999	Alzheimer Disease	Pre-Eclampsia
61	298	0,996356216	Carcinoma, Pancreatic Ductal	Osteosarcoma
250	323	0,996355729	Melanoma	Pre-Eclampsia
67	110	0,996352918	Cardiomyopathy, Hypertrophic	Diabetes Mellitus, Type 2
211	365	0,99635221	Leukemia, Lymphocytic, Acute	Stomach Neoplasms
213	262	0,996351734	Leukemia, Myelocytic, Acute	Multiple Myeloma
5	249	0,996350688	Adrenocortical Carcinoma	Medulloblastoma
213	288	0,996348624	Leukemia, Myelocytic, Acute	Obesity
229	386	0,996345999	Lung Neoplasms	Uterine Cervical Neoplasms
5	33	0,996343663	Adrenocortical Carcinoma	Atrial Fibrillation
8	271	0,996342161	Alzheimer Disease	Myocardial Infarction
129	323	0,996337601	Esophageal Neoplasms	Pre-Eclampsia
61	271	0,996333322	Carcinoma, Pancreatic Ductal	Myocardial Infarction
67	386	0,996332529	Cardiomyopathy, Hypertrophic	Uterine Cervical Neoplasms
62	229	0,996332172	Carcinoma, Renal Cell	Lung Neoplasms
213	282	0,996329847	Leukemia, Myelocytic, Acute	Neuroblastoma
149	271	0,996328218	Glioma	Myocardial Infarction
288	342	0,996327973	Obesity	Retinoblastoma
62	250	0,996327878	Carcinoma, Renal Cell	Melanoma
250	292	0,996322443	Melanoma	Osteoarthritis
229	329	0,99632187	Lung Neoplasms	Prostatic Neoplasms
51	149	0,996320993	Breast Neoplasms	Glioma
149	167	0,99632025	Glioma	Hepatitis B
282	365	0,996312044	Neuroblastoma	Stomach Neoplasms
209	249	0,99631116	Leukemia, B-Cell, Chronic	Medulloblastoma
149	385	0,99631014	Glioma	Urinary Bladder Neoplasms
5	128	0,996309518	Adrenocortical Carcinoma	Epilepsy

5	129	0,996307821	Adrenocortical Carcinoma	Esophageal Neoplasms
149	330	0,996304861	Glioma	Psoriasis
51	323	0,996299862	Breast Neoplasms	Pre-Eclampsia
215	229	0,996296648	Leukemia, Myeloid, Chronic	Lung Neoplasms
61	215	0,996295517	Carcinoma, Pancreatic Ductal	Leukemia, Myeloid, Chronic
124	229	0,996295394	Endometriosis	Lung Neoplasms
168	365	0,996284322	Hepatitis C	Stomach Neoplasms
213	350	0,996276009	Leukemia, Myelocytic, Acute	Sarcoma, Kaposi
168	323	0,996271468	Hepatitis C	Pre-Eclampsia
67	129	0,99627124	Cardiomyopathy, Hypertrophic	Esophageal Neoplasms
61	282	0,996268519	Carcinoma, Pancreatic Ductal	Neuroblastoma
84	365	0,996268422	Colitis, Ulcerative	Stomach Neoplasms
168	271	0,996264028	Hepatitis C	Myocardial Infarction
33	282	0,996258841	Atrial Fibrillation	Neuroblastoma
213	271	0,996256947	Leukemia, Myelocytic, Acute	Myocardial Infarction
209	250	0,996250538	Leukemia, B-Cell, Chronic	Melanoma
29	58	0,99624877	Arthritis, Rheumatoid	Carcinoma, Hepatocellular
8	129	0,996245748	Alzheimer Disease	Esophageal Neoplasms
62	329	0,996245173	Carcinoma, Renal Cell	Prostatic Neoplasms
149	230	0,996240797	Glioma	Lupus Erythematosus, Systemic
33	58	0,996239883	Atrial Fibrillation	Carcinoma, Hepatocellular
108	168	0,996236928	Diabetes Mellitus	Hepatitis C
209	262	0,996230811	Leukemia, B-Cell, Chronic	Multiple Myeloma
91	149	0,99623029	Coronary artery disease	Glioma
209	298	0,996225308	Leukemia, B-Cell, Chronic	Osteosarcoma
292	386	0,996221745	Osteoarthritis	Uterine Cervical Neoplasms
62	209	0,996219915	Carcinoma, Renal Cell	Leukemia, B-Cell, Chronic
32	61	0,996216275	Atherosclerosis	Carcinoma, Pancreatic Ductal
33	149	0,996210222	Atrial Fibrillation	Glioma
211	298	0,996208812	Leukemia, Lymphocytic, Acute	Osteosarcoma
58	84	0,996208629	Carcinoma, Hepatocellular	Colitis, Ulcerative
110	346	0,996206812	Diabetes Mellitus, Type 2	Salivary Gland Neoplasms
61	329	0,996205976	Carcinoma, Pancreatic Ductal	Prostatic Neoplasms
5	323	0,996204902	Adrenocortical Carcinoma	Pre-Eclampsia
149	384	0,996203514	Glioma	Tuberculosis, Pulmonary
250	384	0,996203415	Melanoma	Tuberculosis, Pulmonary
61	67	0,996202822	Carcinoma, Pancreatic Ductal	Cardiomyopathy, Hypertrophic
58	129	0,996202672	Carcinoma, Hepatocellular	Esophageal Neoplasms
61	129	0,996201783	Carcinoma, Pancreatic Ductal	Esophageal Neoplasms
250	271	0,996200987	Melanoma	Myocardial Infarction
58	235	0,996199026	Carcinoma, Hepatocellular	Lymphoma
8	288	0,996196713	Alzheimer Disease	Obesity
5	209	0,996193633	Adrenocortical Carcinoma	Leukemia, B-Cell, Chronic
58	384	0,99619222	Carcinoma, Hepatocellular	Tuberculosis, Pulmonary
58	288	0,996189441	Carcinoma, Hepatocellular	Obesity
8	51	0,996185477	Alzheimer Disease	Breast Neoplasms
91	110	0,996184305	Coronary artery disease	Diabetes Mellitus, Type 2
209	229	0,996183625	Leukemia, B-Cell, Chronic	Lung Neoplasms
250	346	0,996179972	Melanoma	Salivary Gland Neoplasms
8	230	0,996174314	Alzheimer Disease	Lupus Erythematosus, Systemic
32	149	0,996173637	Atherosclerosis	Glioma
67	271	0,996173604	Cardiomyopathy, Hypertrophic	Myocardial Infarction
110	256	0,996171273	Diabetes Mellitus, Type 2	Metabolic Syndrome X
211	229	0,996168479	Leukemia, Lymphocytic, Acute	Lung Neoplasms
108	323	0,996167335	Diabetes Mellitus	Pre-Eclampsia
58	153	0,996157818	Carcinoma, Hepatocellular	Gout
110	323	0,99615584	Diabetes Mellitus, Type 2	Pre-Eclampsia
33	110	0,9961554	Atrial Fibrillation	Diabetes Mellitus, Type 2
84	229	0,996152912	Colitis, Ulcerative	Lung Neoplasms
62	129	0,996152884	Carcinoma, Renal Cell	Esophageal Neoplasms
91	229	0,99615195	Coronary artery disease	Lung Neoplasms
8	235	0,996151825	Alzheimer Disease	Lymphoma
5	235	0,996151095	Adrenocortical Carcinoma	Lymphoma
167	386	0,996149359	Hepatitis B	Uterine Cervical Neoplasms
84	288	0,996148932	Colitis, Ulcerative	Obesity
235	323	0,996147023	Lymphoma	Pre-Eclampsia
8	249	0,996146694	Alzheimer Disease	Medulloblastoma
229	323	0,99614666	Lung Neoplasms	Pre-Eclampsia
213	346	0,996146623	Leukemia, Myelocytic, Acute	Salivary Gland Neoplasms

211	386	0,99614658	Leukemia, Lymphocytic, Acute	Uterine Cervical Neoplasms
62	269	0,996146076	Carcinoma, Renal Cell	Myelodysplastic Syndromes
230	235	0,996145958	Lupus Erythematosus, Systemic	Lymphoma
182	229	0,996142272	Hypertension	Lung Neoplasms
124	249	0,996140776	Endometriosis	Medulloblastoma
110	288	0,996134954	Diabetes Mellitus, Type 2	Obesity
51	167	0,996133826	Breast Neoplasms	Hepatitis B
168	211	0,996133638	Hepatitis C	Leukemia, Lymphocytic, Acute
235	365	0,996132778	Lymphoma	Stomach Neoplasms
167	209	0,996132654	Hepatitis B	Leukemia, B-Cell, Chronic
5	215	0,996132391	Adrenocortical Carcinoma	Leukemia, Myeloid, Chronic
61	62	0,996127754	Carcinoma, Pancreatic Ductal	Carcinoma, Renal Cell
124	149	0,996124424	Endometriosis	Glioma
5	271	0,996115705	Adrenocortical Carcinoma	Myocardial Infarction
67	350	0,99611334	Cardiomyopathy, Hypertrophic	Sarcoma, Kaposi
32	213	0,996113217	Atherosclerosis	Leukemia, Myelocytic, Acute
30	229	0,996112235	Asthma	Lung Neoplasms
29	229	0,996111678	Arthritis, Rheumatoid	Lung Neoplasms
182	298	0,99611034	Hypertension	Osteosarcoma
5	211	0,996109975	Adrenocortical Carcinoma	Leukemia, Lymphocytic, Acute
5	51	0,996106965	Adrenocortical Carcinoma	Breast Neoplasms
61	235	0,996104291	Carcinoma, Pancreatic Ductal	Lymphoma
209	271	0,996102037	Leukemia, B-Cell, Chronic	Myocardial Infarction
209	386	0,996101444	Leukemia, B-Cell, Chronic	Uterine Cervical Neoplasms
213	266	0,996100064	Leukemia, Myelocytic, Acute	Muscular Dystrophy, Duchenne
235	288	0,99609369	Lymphoma	Obesity
62	302	0,996092061	Carcinoma, Renal Cell	Ovarian Neoplasms
211	213	0,996086707	Leukemia, Lymphocytic, Acute	Leukemia, Myelocytic, Acute
58	182	0,99608578	Carcinoma, Hepatocellular	Hypertension
8	110	0,996085367	Alzheimer Disease	Diabetes Mellitus, Type 2
215	365	0,996084856	Leukemia, Myeloid, Chronic	Stomach Neoplasms
302	365	0,996083769	Ovarian Neoplasms	Stomach Neoplasms
229	271	0,996081861	Lung Neoplasms	Myocardial Infarction
168	329	0,996079438	Hepatitis C	Prostatic Neoplasms
167	365	0,996079396	Hepatitis B	Stomach Neoplasms
67	298	0,996078082	Cardiomyopathy, Hypertrophic	Osteosarcoma
262	323	0,996076106	Multiple Myeloma	Pre-Eclampsia
5	84	0,996073658	Adrenocortical Carcinoma	Colitis, Ulcerative
213	222	0,996070429	Leukemia, Myelocytic, Acute	Liver Cirrhosis
256	386	0,996069921	Metabolic Syndrome X	Uterine Cervical Neoplasms
298	334	0,996065373	Osteosarcoma	Pulmonary Fibrosis
271	386	0,99606501	Myocardial Infarction	Uterine Cervical Neoplasms
229	350	0,996061966	Lung Neoplasms	Sarcoma, Kaposi
32	250	0,996061845	Atherosclerosis	Melanoma
110	316	0,996060431	Diabetes Mellitus, Type 2	Pituitary Neoplasms
84	298	0,996052311	Colitis, Ulcerative	Osteosarcoma
229	249	0,996050631	Lung Neoplasms	Medulloblastoma
8	385	0,996047919	Alzheimer Disease	Urinary Bladder Neoplasms
168	386	0,996045576	Hepatitis C	Uterine Cervical Neoplasms
165	249	0,996043609	Hematologic Neoplasms	Medulloblastoma
23	58	0,996041517	Aortic Valve Stenosis	Carcinoma, Hepatocellular
129	209	0,99604148	Esophageal Neoplasms	Leukemia, B-Cell, Chronic
288	330	0,996040124	Obesity	Psoriasis
51	129	0,996037548	Breast Neoplasms	Esophageal Neoplasms
91	213	0,996031247	Coronary artery disease	Leukemia, Myelocytic, Acute
67	249	0,996030879	Cardiomyopathy, Hypertrophic	Medulloblastoma
62	168	0,996030751	Carcinoma, Renal Cell	Hepatitis C
8	168	0,99603069	Alzheimer Disease	Hepatitis C
124	323	0,996030578	Endometriosis	Pre-Eclampsia
323	329	0,996028696	Pre-Eclampsia	Prostatic Neoplasms
149	262	0,996027317	Glioma	Multiple Myeloma
129	386	0,99602416	Esophageal Neoplasms	Uterine Cervical Neoplasms
250	302	0,99602341	Melanoma	Ovarian Neoplasms
108	282	0,996023322	Diabetes Mellitus	Neuroblastoma
86	213	0,996020783	Colorectal Neoplasms	Leukemia, Myelocytic, Acute
129	250	0,996019993	Esophageal Neoplasms	Melanoma
29	365	0,996015536	Arthritis, Rheumatoid	Stomach Neoplasms
323	384	0,996015469	Pre-Eclampsia	Tuberculosis, Pulmonary
168	292	0,996011607	Hepatitis C	Osteoarthritis

167	235	0,996009437	Hepatitis B	Lymphoma
62	323	0,996005653	Carcinoma, Renal Cell	Pre-Eclampsia
5	292	0,99600513	Adrenocortical Carcinoma	Osteoarthritis
249	271	0,99600401	Medulloblastoma	Myocardial Infarction
29	213	0,996001257	Arthritis, Rheumatoid	Leukemia, Myelocytic, Acute
213	230	0,996001069	Leukemia, Myelocytic, Acute	Lupus Erythematosus, Systemic
128	213	0,995999713	Epilepsy	Leukemia, Myelocytic, Acute
182	365	0,995997038	Hypertension	Stomach Neoplasms
215	298	0,995995396	Leukemia, Myeloid, Chronic	Osteosarcoma
86	149	0,99599344	Colorectal Neoplasms	Glioma
58	124	0,995993196	Carcinoma, Hepatocellular	Endometriosis
51	61	0,995991811	Breast Neoplasms	Carcinoma, Pancreatic Ductal
84	386	0,995988625	Colitis, Ulcerative	Uterine Cervical Neoplasms
33	298	0,99598667	Atrial Fibrillation	Osteosarcoma
5	108	0,995981164	Adrenocortical Carcinoma	Diabetes Mellitus
229	288	0,995980748	Lung Neoplasms	Obesity
67	167	0,995979979	Cardiomyopathy, Hypertrophic	Hepatitis B
225	298	0,995978944	Liver Neoplasms	Osteosarcoma
124	282	0,99597809	Endometriosis	Neuroblastoma
168	249	0,995977675	Hepatitis C	Medulloblastoma
67	365	0,995976192	Cardiomyopathy, Hypertrophic	Stomach Neoplasms
110	262	0,995976121	Diabetes Mellitus, Type 2	Multiple Myeloma
58	385	0,995975772	Carcinoma, Hepatocellular	Urinary Bladder Neoplasms
269	298	0,995974562	Myelodysplastic Syndromes	Osteosarcoma
23	250	0,995972842	Aortic Valve Stenosis	Melanoma
51	250	0,995971494	Breast Neoplasms	Melanoma
5	86	0,995969875	Adrenocortical Carcinoma	Colorectal Neoplasms
8	215	0,995969578	Alzheimer Disease	Leukemia, Myeloid, Chronic
5	350	0,995965684	Adrenocortical Carcinoma	Sarcoma, Kaposi
229	385	0,995962127	Lung Neoplasms	Urinary Bladder Neoplasms
108	271	0,995957939	Diabetes Mellitus	Myocardial Infarction
129	365	0,995955709	Esophageal Neoplasms	Stomach Neoplasms
262	365	0,995954674	Multiple Myeloma	Stomach Neoplasms
32	209	0,995953542	Atherosclerosis	Leukemia, B-Cell, Chronic
29	149	0,995951757	Arthritis, Rheumatoid	Glioma
51	282	0,995951287	Breast Neoplasms	Neuroblastoma
62	282	0,995951158	Carcinoma, Renal Cell	Neuroblastoma
91	250	0,995950079	Coronary artery disease	Melanoma
149	215	0,995948831	Glioma	Leukemia, Myeloid, Chronic
86	298	0,99594378	Colorectal Neoplasms	Osteosarcoma
282	292	0,995938701	Neuroblastoma	Osteoarthritis
229	230	0,995938645	Lung Neoplasms	Lupus Erythematosus, Systemic
230	250	0,995936812	Lupus Erythematosus, Systemic	Melanoma
149	292	0,995935832	Glioma	Osteoarthritis
215	292	0,995929917	Leukemia, Myeloid, Chronic	Osteoarthritis
182	215	0,995928191	Hypertension	Leukemia, Myeloid, Chronic
271	365	0,995924725	Myocardial Infarction	Stomach Neoplasms
84	213	0,995924409	Colitis, Ulcerative	Leukemia, Myelocytic, Acute
58	329	0,995923768	Carcinoma, Hepatocellular	Prostatic Neoplasms
58	266	0,995923125	Carcinoma, Hepatocellular	Muscular Dystrophy, Duchenne
229	308	0,995921359	Lung Neoplasms	Parkinson Disease
249	292	0,99591744	Medulloblastoma	Osteoarthritis
250	288	0,995916902	Melanoma	Obesity
8	236	0,995915832	Alzheimer Disease	Lymphoma, B-Cell
329	386	0,995915188	Prostatic Neoplasms	Uterine Cervical Neoplasms
229	330	0,995913772	Lung Neoplasms	Psoriasis
84	182	0,995911758	Colitis, Ulcerative	Hypertension
168	235	0,995909078	Hepatitis C	Lymphoma
350	386	0,995906738	Sarcoma, Kaposi	Uterine Cervical Neoplasms
62	249	0,995904591	Carcinoma, Renal Cell	Medulloblastoma
32	168	0,995904379	Atherosclerosis	Hepatitis C
167	298	0,995904179	Hepatitis B	Osteosarcoma
58	238	0,995903285	Carcinoma, Hepatocellular	Lymphoma, Large-Cell, Diffuse
61	302	0,995902532	Carcinoma, Pancreatic Ductal	Ovarian Neoplasms
149	350	0,995901511	Glioma	Sarcoma, Kaposi
167	323	0,995900168	Hepatitis B	Pre-Eclampsia
58	211	0,995899137	Carcinoma, Hepatocellular	Leukemia, Lymphocytic, Acute
4	229	0,995891941	Adrenal Cortex Neoplasms	Lung Neoplasms
67	329	0,995891137	Cardiomyopathy, Hypertrophic	Prostatic Neoplasms

263	365	0,995888302	Multiple Sclerosis	Stomach Neoplasms
182	250	0,995886116	Hypertension	Melanoma
32	58	0,99588553	Atherosclerosis	Carcinoma, Hepatocellular
168	182	0,995885318	Hepatitis C	Hypertension
32	323	0,995885218	Atherosclerosis	Pre-Eclampsia
124	215	0,995884795	Endometriosis	Leukemia, Myeloid, Chronic
33	229	0,995884114	Atrial Fibrillation	Lung Neoplasms
209	385	0,995884063	Leukemia, B-Cell, Chronic	Urinary Bladder Neoplasms
110	182	0,995883789	Diabetes Mellitus, Type 2	Hypertension
176	213	0,995883095	Hodgkin Disease	Leukemia, Myelocytic, Acute
4	58	0,995881001	Adrenal Cortex Neoplasms	Carcinoma, Hepatocellular
165	365	0,995880591	Hematologic Neoplasms	Stomach Neoplasms
8	308	0,995876101	Alzheimer Disease	Parkinson Disease
33	250	0,995875522	Atrial Fibrillation	Melanoma
108	365	0,995872384	Diabetes Mellitus	Stomach Neoplasms
128	250	0,995869812	Epilepsy	Melanoma
168	334	0,995869561	Hepatitis C	Pulmonary Fibrosis
5	262	0,995869279	Adrenocortical Carcinoma	Multiple Myeloma
32	271	0,995867831	Atherosclerosis	Myocardial Infarction
84	250	0,995867733	Colitis, Ulcerative	Melanoma
149	288	0,995867491	Glioma	Obesity
168	262	0,995866016	Hepatitis C	Multiple Myeloma
229	235	0,995861008	Lung Neoplasms	Lymphoma
29	182	0,995859678	Arthritis, Rheumatoid	Hypertension
30	58	0,995856404	Asthma	Carcinoma, Hepatocellular
58	86	0,99585507	Carcinoma, Hepatocellular	Colorectal Neoplasms
176	209	0,99585395	Hodgkin Disease	Leukemia, B-Cell, Chronic
32	215	0,995853736	Atherosclerosis	Leukemia, Myeloid, Chronic
149	342	0,995852816	Glioma	Retinoblastoma
67	168	0,995849997	Cardiomyopathy, Hypertrophic	Hepatitis C
250	329	0,995847279	Melanoma	Prostatic Neoplasms
84	230	0,995844143	Colitis, Ulcerative	Lupus Erythematosus, Systemic
168	288	0,99584395	Hepatitis C	Obesity
149	266	0,995841528	Glioma	Muscular Dystrophy, Duchenne
67	215	0,995840594	Cardiomyopathy, Hypertrophic	Leukemia, Myeloid, Chronic
110	128	0,995837102	Diabetes Mellitus, Type 2	Epilepsy
4	365	0,995836982	Adrenal Cortex Neoplasms	Stomach Neoplasms
288	386	0,995836188	Obesity	Uterine Cervical Neoplasms
182	262	0,995832891	Hypertension	Multiple Myeloma
302	330	0,995828452	Ovarian Neoplasms	Psoriasis
209	292	0,995827681	Leukemia, B-Cell, Chronic	Osteoarthritis
67	84	0,995825971	Cardiomyopathy, Hypertrophic	Colitis, Ulcerative
33	365	0,995824345	Atrial Fibrillation	Stomach Neoplasms
8	350	0,995824169	Alzheimer Disease	Sarcoma, Kaposi
33	213	0,995823765	Atrial Fibrillation	Leukemia, Myelocytic, Acute
209	222	0,995822288	Leukemia, B-Cell, Chronic	Liver Cirrhosis
110	235	0,995821341	Diabetes Mellitus, Type 2	Lymphoma
215	271	0,995820503	Leukemia, Myeloid, Chronic	Myocardial Infarction
168	266	0,995818917	Hepatitis C	Muscular Dystrophy, Duchenne
385	386	0,995817924	Urinary Bladder Neoplasms	Uterine Cervical Neoplasms
91	386	0,995817233	Coronary artery disease	Uterine Cervical Neoplasms
61	330	0,995816948	Carcinoma, Pancreatic Ductal	Psoriasis
229	262	0,995812765	Lung Neoplasms	Multiple Myeloma
209	235	0,995811139	Leukemia, B-Cell, Chronic	Lymphoma
330	365	0,995810503	Psoriasis	Stomach Neoplasms
61	292	0,995808179	Carcinoma, Pancreatic Ductal	Osteoarthritis
91	249	0,995806138	Coronary artery disease	Medulloblastoma
182	209	0,995804023	Hypertension	Leukemia, B-Cell, Chronic
32	110	0,995798053	Atherosclerosis	Diabetes Mellitus, Type 2
58	262	0,995796493	Carcinoma, Hepatocellular	Multiple Myeloma
8	108	0,995795124	Alzheimer Disease	Diabetes Mellitus
229	302	0,995793877	Lung Neoplasms	Ovarian Neoplasms
33	215	0,995790515	Atrial Fibrillation	Leukemia, Myeloid, Chronic
58	334	0,995790382	Carcinoma, Hepatocellular	Pulmonary Fibrosis
182	213	0,995788303	Hypertension	Leukemia, Myelocytic, Acute
149	308	0,995788037	Glioma	Parkinson Disease
262	292	0,995787918	Multiple Myeloma	Osteoarthritis
29	282	0,995786657	Arthritis, Rheumatoid	Neuroblastoma
86	365	0,995784242	Colorectal Neoplasms	Stomach Neoplasms

33	182	0,99578393	Atrial Fibrillation	Hypertension
67	342	0,995782708	Cardiomyopathy, Hypertrophic	Retinoblastoma
86	110	0,995781272	Colorectal Neoplasms	Diabetes Mellitus, Type 2
5	385	0,995776508	Adrenocortical Carcinoma	Urinary Bladder Neoplasms
168	215	0,995776091	Hepatitis C	Leukemia, Myeloid, Chronic
108	386	0,995774112	Diabetes Mellitus	Uterine Cervical Neoplasms
86	288	0,995768474	Colorectal Neoplasms	Obesity
110	222	0,995768055	Diabetes Mellitus, Type 2	Liver Cirrhosis
29	292	0,995767678	Arthritis, Rheumatoid	Osteoarthritis
298	342	0,995765745	Osteosarcoma	Retinoblastoma
91	282	0,995765618	Coronary artery disease	Neuroblastoma
149	193	0,995764919	Glioma	Influenza, Human
282	323	0,995764275	Neuroblastoma	Pre-Eclampsia
222	229	0,995763602	Liver Cirrhosis	Lung Neoplasms
209	329	0,995763453	Leukemia, B-Cell, Chronic	Prostatic Neoplasms
298	303	0,995763435	Osteosarcoma	Pancreatic Neoplasms
282	288	0,995763186	Neuroblastoma	Obesity
84	215	0,995762097	Colitis, Ulcerative	Leukemia, Myeloid, Chronic
292	365	0,995760764	Osteoarthritis	Stomach Neoplasms
32	229	0,995759991	Atherosclerosis	Lung Neoplasms
67	385	0,995759244	Cardiomyopathy, Hypertrophic	Urinary Bladder Neoplasms
129	329	0,995757599	Esophageal Neoplasms	Prostatic Neoplasms
86	235	0,9957575	Colorectal Neoplasms	Lymphoma
30	167	0,995755528	Asthma	Hepatitis B
51	110	0,995753882	Breast Neoplasms	Diabetes Mellitus, Type 2
5	346	0,995751899	Adrenocortical Carcinoma	Salivary Gland Neoplasms
129	249	0,995750626	Esophageal Neoplasms	Medulloblastoma
110	266	0,995749502	Diabetes Mellitus, Type 2	Muscular Dystrophy, Duchenne
67	282	0,995749211	Cardiomyopathy, Hypertrophic	Neuroblastoma
298	323	0,99574814	Osteosarcoma	Pre-Eclampsia
215	386	0,995746328	Leukemia, Myeloid, Chronic	Uterine Cervical Neoplasms
5	23	0,995745724	Adrenocortical Carcinoma	Aortic Valve Stenosis
262	386	0,995745665	Multiple Myeloma	Uterine Cervical Neoplasms
84	323	0,995744732	Colitis, Ulcerative	Pre-Eclampsia
110	124	0,995744157	Diabetes Mellitus, Type 2	Endometriosis
211	350	0,995743386	Leukemia, Lymphocytic, Acute	Sarcoma, Kaposi
167	292	0,99574261	Hepatitis B	Osteoarthritis
110	292	0,995741421	Diabetes Mellitus, Type 2	Osteoarthritis
84	262	0,995738545	Colitis, Ulcerative	Multiple Myeloma
182	386	0,995738083	Hypertension	Uterine Cervical Neoplasms
209	288	0,995737896	Leukemia, B-Cell, Chronic	Obesity
308	323	0,995735528	Parkinson Disease	Pre-Eclampsia
58	225	0,99573182	Carcinoma, Hepatocellular	Liver Neoplasms
62	235	0,995730847	Carcinoma, Renal Cell	Lymphoma
84	149	0,995730465	Colitis, Ulcerative	Glioma
62	67	0,995729489	Carcinoma, Renal Cell	Cardiomyopathy, Hypertrophic
229	342	0,995728964	Lung Neoplasms	Retinoblastoma
215	350	0,995728444	Leukemia, Myeloid, Chronic	Sarcoma, Kaposi
292	298	0,99572699	Osteoarthritis	Osteosarcoma
23	229	0,99572637	Aortic Valve Stenosis	Lung Neoplasms
5	32	0,995726261	Adrenocortical Carcinoma	Atherosclerosis
129	316	0,995725058	Esophageal Neoplasms	Pituitary Neoplasms
33	271	0,995724991	Atrial Fibrillation	Myocardial Infarction
84	292	0,995723062	Colitis, Ulcerative	Osteoarthritis
302	386	0,995722564	Ovarian Neoplasms	Uterine Cervical Neoplasms
32	235	0,99572071	Atherosclerosis	Lymphoma
249	386	0,995720635	Medulloblastoma	Uterine Cervical Neoplasms
32	282	0,995713606	Atherosclerosis	Neuroblastoma
149	153	0,995713145	Glioma	Gout
67	230	0,995712438	Cardiomyopathy, Hypertrophic	Lupus Erythematosus, Systemic
61	128	0,995712154	Carcinoma, Pancreatic Ductal	Epilepsy
84	329	0,995711945	Colitis, Ulcerative	Prostatic Neoplasms
8	33	0,995711884	Alzheimer Disease	Atrial Fibrillation
230	323	0,995708067	Lupus Erythematosus, Systemic	Pre-Eclampsia
230	288	0,995707307	Lupus Erythematosus, Systemic	Obesity
167	288	0,995705862	Hepatitis B	Obesity
235	385	0,995705725	Lymphoma	Urinary Bladder Neoplasms
91	330	0,99570546	Coronary artery disease	Psoriasis
8	292	0,995705208	Alzheimer Disease	Osteoarthritis

110	329	0,995704377	Diabetes Mellitus, Type 2	Prostatic Neoplasms
84	330	0,995703391	Colitis, Ulcerative	Psoriasis
168	316	0,995702403	Hepatitis C	Pituitary Neoplasms
129	271	0,995701975	Esophageal Neoplasms	Myocardial Infarction
365	384	0,995700217	Stomach Neoplasms	Tuberculosis, Pulmonary
84	353	0,995698989	Colitis, Ulcerative	Schizophrenia
250	330	0,995698692	Melanoma	Psoriasis
168	209	0,995695186	Hepatitis C	Leukemia, B-Cell, Chronic
238	323	0,995695063	Lymphoma, Large-Cell, Diffuse	Pre-Eclampsia
129	167	0,995694216	Esophageal Neoplasms	Hepatitis B
91	211	0,995694152	Coronary artery disease	Leukemia, Lymphocytic, Acute
323	330	0,995693624	Pre-Eclampsia	Psoriasis
209	211	0,995690113	Leukemia, B-Cell, Chronic	Leukemia, Lymphocytic, Acute
149	269	0,995686638	Glioma	Myelodysplastic Syndromes
23	29	0,995684342	Aortic Valve Stenosis	Arthritis, Rheumatoid
229	292	0,995683542	Lung Neoplasms	Osteoarthritis
129	168	0,995678325	Esophageal Neoplasms	Hepatitis C
235	271	0,995678174	Lymphoma	Myocardial Infarction
225	386	0,995672988	Liver Neoplasms	Uterine Cervical Neoplasms
67	211	0,995669591	Cardiomyopathy, Hypertrophic	Leukemia, Lymphocytic, Acute
51	330	0,995666271	Breast Neoplasms	Psoriasis
110	302	0,995664362	Diabetes Mellitus, Type 2	Ovarian Neoplasms
229	266	0,995664121	Lung Neoplasms	Muscular Dystrophy, Duchenne
108	250	0,995662861	Diabetes Mellitus	Melanoma
67	288	0,995662687	Cardiomyopathy, Hypertrophic	Obesity
262	329	0,995659985	Multiple Myeloma	Prostatic Neoplasms
129	298	0,995659466	Esophageal Neoplasms	Osteosarcoma
91	168	0,995657839	Coronary artery disease	Hepatitis C
149	182	0,995657278	Glioma	Hypertension
249	288	0,995656941	Medulloblastoma	Obesity
61	385	0,995655494	Carcinoma, Pancreatic Ductal	Urinary Bladder Neoplasms
23	67	0,995655476	Aortic Valve Stenosis	Cardiomyopathy, Hypertrophic
29	215	0,995655107	Arthritis, Rheumatoid	Leukemia, Myeloid, Chronic
58	346	0,995654546	Carcinoma, Hepatocellular	Salivary Gland Neoplasms
108	213	0,995654353	Diabetes Mellitus	Leukemia, Myelocytic, Acute
271	288	0,995652425	Myocardial Infarction	Obesity
167	230	0,995652277	Hepatitis B	Lupus Erythematosus, Systemic
8	342	0,995651434	Alzheimer Disease	Retinoblastoma
61	84	0,995651107	Carcinoma, Pancreatic Ductal	Colitis, Ulcerative
215	346	0,99565076	Leukemia, Myeloid, Chronic	Salivary Gland Neoplasms
330	386	0,99564488	Psoriasis	Uterine Cervical Neoplasms
29	250	0,995643674	Arthritis, Rheumatoid	Melanoma
271	282	0,995640353	Myocardial Infarction	Neuroblastoma
308	385	0,995640276	Parkinson Disease	Urinary Bladder Neoplasms
61	211	0,99563802	Carcinoma, Pancreatic Ductal	Leukemia, Lymphocytic, Acute
5	329	0,995637783	Adrenocortical Carcinoma	Prostatic Neoplasms
61	256	0,995631566	Carcinoma, Pancreatic Ductal	Metabolic Syndrome X
32	365	0,995631304	Atherosclerosis	Stomach Neoplasms
33	167	0,995630355	Atrial Fibrillation	Hepatitis B
302	323	0,995624475	Ovarian Neoplasms	Pre-Eclampsia
167	215	0,995623681	Hepatitis B	Leukemia, Myeloid, Chronic
250	334	0,995623335	Melanoma	Pulmonary Fibrosis
58	222	0,995621255	Carcinoma, Hepatocellular	Liver Cirrhosis
211	256	0,995620798	Leukemia, Lymphocytic, Acute	Metabolic Syndrome X
282	302	0,99562035	Neuroblastoma	Ovarian Neoplasms
271	385	0,995619318	Myocardial Infarction	Urinary Bladder Neoplasms
182	292	0,995616897	Hypertension	Osteoarthritis
84	249	0,995615987	Colitis, Ulcerative	Medulloblastoma
23	292	0,995614932	Aortic Valve Stenosis	Osteoarthritis
282	346	0,995614426	Neuroblastoma	Salivary Gland Neoplasms
110	167	0,995611118	Diabetes Mellitus, Type 2	Hepatitis B
282	316	0,995610673	Neuroblastoma	Pituitary Neoplasms
32	211	0,995610377	Atherosclerosis	Leukemia, Lymphocytic, Acute
215	323	0,995607414	Leukemia, Myeloid, Chronic	Pre-Eclampsia
108	110	0,995606703	Diabetes Mellitus	Diabetes Mellitus, Type 2
262	298	0,995606146	Multiple Myeloma	Osteosarcoma
30	215	0,995603527	Asthma	Leukemia, Myeloid, Chronic
209	215	0,995603439	Leukemia, B-Cell, Chronic	Leukemia, Myeloid, Chronic
384	386	0,995598451	Tuberculosis, Pulmonary	Uterine Cervical Neoplasms

215	342	0,995598056	Leukemia, Myeloid, Chronic	Retinoblastoma
288	385	0,995596048	Obesity	Urinary Bladder Neoplasms
84	110	0,995596034	Colitis, Ulcerative	Diabetes Mellitus, Type 2
5	266	0,99559447	Adrenocortical Carcinoma	Muscular Dystrophy, Duchenne
61	230	0,995594385	Carcinoma, Pancreatic Ductal	Lupus Erythematosus, Systemic
149	303	0,995591951	Glioma	Pancreatic Neoplasms
32	385	0,995589961	Atherosclerosis	Urinary Bladder Neoplasms
67	262	0,99558867	Cardiomyopathy, Hypertrophic	Multiple Myeloma
230	386	0,995587634	Lupus Erythematosus, Systemic	Uterine Cervical Neoplasms
149	165	0,995587165	Glioma	Hematologic Neoplasms
282	308	0,995585	Neuroblastoma	Parkinson Disease
86	282	0,995584971	Colorectal Neoplasms	Neuroblastoma
61	222	0,995584044	Carcinoma, Pancreatic Ductal	Liver Cirrhosis
58	302	0,995578169	Carcinoma, Hepatocellular	Ovarian Neoplasms
5	263	0,995577338	Adrenocortical Carcinoma	Multiple Sclerosis
110	263	0,99557623	Diabetes Mellitus, Type 2	Multiple Sclerosis
51	271	0,995573412	Breast Neoplasms	Myocardial Infarction
8	384	0,995571762	Alzheimer Disease	Tuberculosis, Pulmonary
250	262	0,995570298	Melanoma	Multiple Myeloma
149	225	0,995570209	Glioma	Liver Neoplasms
62	303	0,99556737	Carcinoma, Renal Cell	Pancreatic Neoplasms
108	288	0,995567365	Diabetes Mellitus	Obesity
262	350	0,995565652	Multiple Myeloma	Sarcoma, Kaposi
61	342	0,995562474	Carcinoma, Pancreatic Ductal	Retinoblastoma
84	168	0,995562365	Colitis, Ulcerative	Hepatitis C
4	62	0,995561599	Adrenal Cortex Neoplasms	Carcinoma, Renal Cell
282	386	0,995560097	Neuroblastoma	Uterine Cervical Neoplasms
86	168	0,995556901	Colorectal Neoplasms	Hepatitis C
110	384	0,995554556	Diabetes Mellitus, Type 2	Tuberculosis, Pulmonary
108	229	0,99555295	Diabetes Mellitus	Lung Neoplasms
230	365	0,995552935	Lupus Erythematosus, Systemic	Stomach Neoplasms
63	213	0,995548669	Carcinoma, Squamous Cell	Leukemia, Myelocytic, Acute
58	176	0,995547314	Carcinoma, Hepatocellular	Hodgkin Disease
282	342	0,995547055	Neuroblastoma	Retinoblastoma
129	385	0,995545969	Esophageal Neoplasms	Urinary Bladder Neoplasms
29	323	0,99554536	Arthritis, Rheumatoid	Pre-Eclampsia
67	330	0,995545164	Cardiomyopathy, Hypertrophic	Psoriasis
250	342	0,995542326	Melanoma	Retinoblastoma
23	249	0,995537322	Aortic Valve Stenosis	Medulloblastoma
110	151	0,995536093	Diabetes Mellitus, Type 2	Glomerulonephritis, IGA
33	84	0,995535666	Atrial Fibrillation	Colitis, Ulcerative
29	249	0,995535173	Arthritis, Rheumatoid	Medulloblastoma
33	168	0,995534422	Atrial Fibrillation	Hepatitis C
288	350	0,995532946	Obesity	Sarcoma, Kaposi
62	91	0,995528595	Carcinoma, Renal Cell	Coronary artery disease
32	62	0,995527154	Atherosclerosis	Carcinoma, Renal Cell
215	288	0,995526464	Leukemia, Myeloid, Chronic	Obesity
62	86	0,995524264	Carcinoma, Renal Cell	Colorectal Neoplasms
235	329	0,995523639	Lymphoma	Prostatic Neoplasms
222	292	0,995520912	Liver Cirrhosis	Osteoarthritis
308	365	0,99551941	Parkinson Disease	Stomach Neoplasms
213	334	0,995517474	Leukemia, Myelocytic, Acute	Pulmonary Fibrosis
262	282	0,99551638	Multiple Myeloma	Neuroblastoma
211	288	0,995516281	Leukemia, Lymphocytic, Acute	Obesity
238	250	0,99551413	Lymphoma, Large-Cell, Diffuse	Melanoma
249	262	0,995513406	Medulloblastoma	Multiple Myeloma
86	229	0,995512895	Colorectal Neoplasms	Lung Neoplasms
108	230	0,995512651	Diabetes Mellitus	Lupus Erythematosus, Systemic
225	330	0,99551197	Liver Neoplasms	Psoriasis
235	249	0,995511771	Lymphoma	Medulloblastoma
211	271	0,995509823	Leukemia, Lymphocytic, Acute	Myocardial Infarction
62	262	0,995509646	Carcinoma, Renal Cell	Multiple Myeloma
230	342	0,995508992	Lupus Erythematosus, Systemic	Retinoblastoma
29	84	0,995508189	Arthritis, Rheumatoid	Colitis, Ulcerative
32	167	0,99550804	Atherosclerosis	Hepatitis B
8	32	0,995503239	Alzheimer Disease	Atherosclerosis
8	330	0,995500391	Alzheimer Disease	Psoriasis
5	167	0,995500251	Adrenocortical Carcinoma	Hepatitis B
8	29	0,995499021	Alzheimer Disease	Arthritis, Rheumatoid

250	266	0,995497859	Melanoma	Muscular Dystrophy, Duchenne
167	271	0,995494021	Hepatitis B	Myocardial Infarction
222	298	0,995492773	Liver Cirrhosis	Osteosarcoma
84	342	0,995489127	Colitis, Ulcerative	Retinoblastoma
91	323	0,995488632	Coronary artery disease	Pre-Eclampsia
209	282	0,995483728	Leukemia, B-Cell, Chronic	Neuroblastoma
23	211	0,995482836	Aortic Valve Stenosis	Leukemia, Lymphocytic, Acute
298	302	0,9954828	Osteosarcoma	Ovarian Neoplasms
5	225	0,995480369	Adrenocortical Carcinoma	Liver Neoplasms
61	262	0,995480194	Carcinoma, Pancreatic Ductal	Multiple Myeloma
129	302	0,995480033	Esophageal Neoplasms	Ovarian Neoplasms
128	271	0,995478041	Epilepsy	Myocardial Infarction
51	386	0,995477817	Breast Neoplasms	Uterine Cervical Neoplasms
8	238	0,995476021	Alzheimer Disease	Lymphoma, Large-Cell, Diffuse
108	292	0,995475623	Diabetes Mellitus	Osteoarthritis
298	350	0,99547561	Osteosarcoma	Sarcoma, Kaposi
58	342	0,995474915	Carcinoma, Hepatocellular	Retinoblastoma
5	303	0,995472148	Adrenocortical Carcinoma	Pancreatic Neoplasms
342	365	0,995471156	Retinoblastoma	Stomach Neoplasms
182	329	0,99547033	Hypertension	Prostatic Neoplasms
298	384	0,995470115	Osteosarcoma	Tuberculosis, Pulmonary
249	323	0,995470037	Medulloblastoma	Pre-Eclampsia
5	384	0,995469732	Adrenocortical Carcinoma	Tuberculosis, Pulmonary
225	365	0,995468763	Liver Neoplasms	Stomach Neoplasms
149	353	0,99546708	Glioma	Schizophrenia
33	288	0,995466018	Atrial Fibrillation	Obesity
334	386	0,995464837	Pulmonary Fibrosis	Uterine Cervical Neoplasms
5	230	0,995464698	Adrenocortical Carcinoma	Lupus Erythematosus, Systemic
149	334	0,995461994	Glioma	Pulmonary Fibrosis
213	238	0,995459897	Leukemia, Myelocytic, Acute	Lymphoma, Large-Cell, Diffuse
32	91	0,99545869	Atherosclerosis	Coronary artery disease
23	262	0,995456923	Aortic Valve Stenosis	Multiple Myeloma
167	282	0,99545685	Hepatitis B	Neuroblastoma
176	229	0,995453617	Hodgkin Disease	Lung Neoplasms
5	342	0,995452608	Adrenocortical Carcinoma	Retinoblastoma
165	168	0,995452396	Hematologic Neoplasms	Hepatitis C
229	316	0,995452093	Lung Neoplasms	Pituitary Neoplasms
262	346	0,995448356	Multiple Myeloma	Salivary Gland Neoplasms
4	386	0,995448254	Adrenal Cortex Neoplasms	Uterine Cervical Neoplasms
61	209	0,995446221	Carcinoma, Pancreatic Ductal	Leukemia, B-Cell, Chronic
51	329	0,995445023	Breast Neoplasms	Prostatic Neoplasms
30	250	0,995444717	Asthma	Melanoma
61	124	0,995444087	Carcinoma, Pancreatic Ductal	Endometriosis
32	51	0,995443873	Atherosclerosis	Breast Neoplasms
167	182	0,995443527	Hepatitis B	Hypertension
62	346	0,995442573	Carcinoma, Renal Cell	Salivary Gland Neoplasms
33	329	0,995442348	Atrial Fibrillation	Prostatic Neoplasms
51	249	0,995440323	Breast Neoplasms	Medulloblastoma
229	353	0,995439559	Lung Neoplasms	Schizophrenia
176	365	0,995439337	Hodgkin Disease	Stomach Neoplasms
61	350	0,995438471	Carcinoma, Pancreatic Ductal	Sarcoma, Kaposi
211	215	0,995438109	Leukemia, Lymphocytic, Acute	Leukemia, Myeloid, Chronic
33	350	0,995437486	Atrial Fibrillation	Sarcoma, Kaposi
32	302	0,99543588	Atherosclerosis	Ovarian Neoplasms
250	385	0,995434259	Melanoma	Urinary Bladder Neoplasms
13	386	0,995433771	Aneurysm	Uterine Cervical Neoplasms
167	350	0,995433372	Hepatitis B	Sarcoma, Kaposi
151	168	0,99543298	Glomerulonephritis, IGA	Hepatitis C
229	346	0,995432301	Lung Neoplasms	Salivary Gland Neoplasms
33	91	0,995431101	Atrial Fibrillation	Coronary artery disease
58	223	0,995430982	Carcinoma, Hepatocellular	Liver Cirrhosis, Biliary
33	86	0,995430061	Atrial Fibrillation	Colorectal Neoplasms
110	225	0,99542747	Diabetes Mellitus, Type 2	Liver Neoplasms
35	386	0,995427452	Autistic Disorder	Uterine Cervical Neoplasms
29	51	0,995427448	Arthritis, Rheumatoid	Breast Neoplasms
32	108	0,995425804	Atherosclerosis	Diabetes Mellitus
165	213	0,995421598	Hematologic Neoplasms	Leukemia, Myelocytic, Acute
61	346	0,995418584	Carcinoma, Pancreatic Ductal	Salivary Gland Neoplasms
5	124	0,995415317	Adrenocortical Carcinoma	Endometriosis

235	282	0,995415273	Lymphoma	Neuroblastoma
51	350	0,99541458	Breast Neoplasms	Sarcoma, Kaposi
67	323	0,995414138	Cardiomyopathy, Hypertrophic	Pre-Eclampsia
91	108	0,9954125	Coronary artery disease	Diabetes Mellitus
330	342	0,995409653	Psoriasis	Retinoblastoma
167	238	0,995408347	Hepatitis B	Lymphoma, Large-Cell, Diffuse
215	222	0,995407187	Leukemia, Myeloid, Chronic	Liver Cirrhosis
5	8	0,995405593	Adrenocortical Carcinoma	Alzheimer Disease
91	235	0,995405367	Coronary artery disease	Lymphoma
151	213	0,995405182	Glomerulonephritis, IGA	Leukemia, Myelocytic, Acute
292	334	0,995404854	Osteoarthritis	Pulmonary Fibrosis
235	292	0,995402669	Lymphoma	Osteoarthritis
8	84	0,995402171	Alzheimer Disease	Colitis, Ulcerative
288	334	0,995397545	Obesity	Pulmonary Fibrosis
51	62	0,995395861	Breast Neoplasms	Carcinoma, Renal Cell
61	108	0,995395541	Carcinoma, Pancreatic Ductal	Diabetes Mellitus
168	263	0,995395271	Hepatitis C	Multiple Sclerosis
29	288	0,995394373	Arthritis, Rheumatoid	Obesity
271	323	0,995393813	Myocardial Infarction	Pre-Eclampsia
51	209	0,99539093	Breast Neoplasms	Leukemia, B-Cell, Chronic
129	288	0,995387843	Esophageal Neoplasms	Obesity
271	384	0,995387722	Myocardial Infarction	Tuberculosis, Pulmonary
168	385	0,995387146	Hepatitis C	Urinary Bladder Neoplasms
108	167	0,995386435	Diabetes Mellitus	Hepatitis B
213	342	0,995385509	Leukemia, Myelocytic, Acute	Retinoblastoma
298	329	0,995385494	Osteosarcoma	Prostatic Neoplasms
29	329	0,995384904	Arthritis, Rheumatoid	Prostatic Neoplasms
51	124	0,995384246	Breast Neoplasms	Endometriosis
250	339	0,995383669	Melanoma	Respiratory Syncytial Virus Infections
91	288	0,99538347	Coronary artery disease	Obesity
149	346	0,995378689	Glioma	Salivary Gland Neoplasms
242	292	0,995377752	Lymphoma, T-Cell	Osteoarthritis
30	182	0,995376492	Asthma	Hypertension
209	342	0,995376384	Leukemia, B-Cell, Chronic	Retinoblastoma
108	350	0,9953761	Diabetes Mellitus	Sarcoma, Kaposi
61	266	0,995375732	Carcinoma, Pancreatic Ductal	Muscular Dystrophy, Duchenne
213	302	0,995375632	Leukemia, Myelocytic, Acute	Ovarian Neoplasms
51	67	0,995374958	Breast Neoplasms	Cardiomyopathy, Hypertrophic
29	110	0,995374882	Arthritis, Rheumatoid	Diabetes Mellitus, Type 2
84	209	0,995372827	Colitis, Ulcerative	Leukemia, B-Cell, Chronic
209	384	0,995372804	Leukemia, B-Cell, Chronic	Tuberculosis, Pulmonary
61	308	0,995372482	Carcinoma, Pancreatic Ductal	Parkinson Disease
58	236	0,995372095	Carcinoma, Hepatocellular	Lymphoma, B-Cell
33	249	0,995371904	Atrial Fibrillation	Medulloblastoma
84	86	0,995367853	Colitis, Ulcerative	Colorectal Neoplasms
298	353	0,995365066	Osteosarcoma	Schizophrenia
282	350	0,995364909	Neuroblastoma	Sarcoma, Kaposi
263	329	0,995364853	Multiple Sclerosis	Prostatic Neoplasms
249	346	0,995363943	Medulloblastoma	Salivary Gland Neoplasms
238	292	0,995363295	Lymphoma, Large-Cell, Diffuse	Osteoarthritis
271	298	0,995362282	Myocardial Infarction	Osteosarcoma
211	292	0,995361765	Leukemia, Lymphocytic, Acute	Osteoarthritis
4	213	0,995361513	Adrenal Cortex Neoplasms	Leukemia, Myelocytic, Acute
32	350	0,995361141	Atherosclerosis	Sarcoma, Kaposi
211	385	0,995360079	Leukemia, Lymphocytic, Acute	Urinary Bladder Neoplasms
266	386	0,995359217	Muscular Dystrophy, Duchenne	Uterine Cervical Neoplasms
129	330	0,995358827	Esophageal Neoplasms	Psoriasis
282	334	0,995355559	Neuroblastoma	Pulmonary Fibrosis
35	58	0,995354329	Autistic Disorder	Carcinoma, Hepatocellular
168	384	0,99535414	Hepatitis C	Tuberculosis, Pulmonary
229	384	0,995353589	Lung Neoplasms	Tuberculosis, Pulmonary
316	342	0,995353002	Pituitary Neoplasms	Retinoblastoma
108	209	0,995352759	Diabetes Mellitus	Leukemia, B-Cell, Chronic
129	346	0,995352097	Esophageal Neoplasms	Salivary Gland Neoplasms
108	249	0,995351713	Diabetes Mellitus	Medulloblastoma
23	124	0,995348912	Aortic Valve Stenosis	Endometriosis
298	330	0,995346908	Osteosarcoma	Psoriasis
61	263	0,995346149	Carcinoma, Pancreatic Ductal	Multiple Sclerosis
62	385	0,995345335	Carcinoma, Renal Cell	Urinary Bladder Neoplasms

4	149	0,995344678	Adrenal Cortex Neoplasms	Glioma
33	323	0,99534411	Atrial Fibrillation	Pre-Eclampsia
209	308	0,995343032	Leukemia, B-Cell, Chronic	Parkinson Disease
236	386	0,995342821	Lymphoma, B-Cell	Uterine Cervical Neoplasms
182	323	0,995342177	Hypertension	Pre-Eclampsia
86	386	0,995339195	Colorectal Neoplasms	Uterine Cervical Neoplasms
167	168	0,995337595	Hepatitis B	Hepatitis C
271	292	0,995335712	Myocardial Infarction	Osteoarthritis
108	129	0,99533535	Diabetes Mellitus	Esophageal Neoplasms
62	84	0,995334667	Carcinoma, Renal Cell	Colitis, Ulcerative
5	182	0,995333883	Adrenocortical Carcinoma	Hypertension
8	124	0,99533266	Alzheimer Disease	Endometriosis
165	298	0,995329109	Hematologic Neoplasms	Osteosarcoma
262	288	0,995326539	Multiple Myeloma	Obesity
209	350	0,99532546	Leukemia, B-Cell, Chronic	Sarcoma, Kaposi
353	386	0,995324952	Schizophrenia	Uterine Cervical Neoplasms
62	330	0,995324567	Carcinoma, Renal Cell	Psoriasis
58	206	0,995323511	Carcinoma, Hepatocellular	Leiomyoma
4	323	0,995323498	Adrenal Cortex Neoplasms	Pre-Eclampsia
23	323	0,995322158	Aortic Valve Stenosis	Pre-Eclampsia
323	334	0,995321826	Pre-Eclampsia	Pulmonary Fibrosis
124	298	0,995319046	Endometriosis	Osteosarcoma
5	334	0,995318356	Adrenocortical Carcinoma	Pulmonary Fibrosis
110	230	0,995317745	Diabetes Mellitus, Type 2	Lupus Erythematosus, Systemic
33	385	0,995316736	Atrial Fibrillation	Urinary Bladder Neoplasms
108	211	0,99531582	Diabetes Mellitus	Leukemia, Lymphocytic, Acute
149	316	0,99531303	Glioma	Pituitary Neoplasms
61	225	0,99531302	Carcinoma, Pancreatic Ductal	Liver Neoplasms
29	129	0,995309249	Arthritis, Rheumatoid	Esophageal Neoplasms
176	215	0,995308939	Hodgkin Disease	Leukemia, Myeloid, Chronic
229	269	0,995306874	Lung Neoplasms	Myelodysplastic Syndromes
129	222	0,995306377	Esophageal Neoplasms	Liver Cirrhosis
51	302	0,995305826	Breast Neoplasms	Ovarian Neoplasms
33	230	0,995304327	Atrial Fibrillation	Lupus Erythematosus, Systemic
8	211	0,995304289	Alzheimer Disease	Leukemia, Lymphocytic, Acute
182	249	0,995303416	Hypertension	Medulloblastoma
230	330	0,995301302	Lupus Erythematosus, Systemic	Psoriasis
29	209	0,995296931	Arthritis, Rheumatoid	Leukemia, B-Cell, Chronic
350	365	0,995296311	Sarcoma, Kaposi	Stomach Neoplasms
167	249	0,995294104	Hepatitis B	Medulloblastoma
225	250	0,995293999	Liver Neoplasms	Melanoma
250	256	0,995293958	Melanoma	Metabolic Syndrome X
62	211	0,99529383	Carcinoma, Renal Cell	Leukemia, Lymphocytic, Acute
168	350	0,995293408	Hepatitis C	Sarcoma, Kaposi
292	350	0,995293352	Osteoarthritis	Sarcoma, Kaposi
51	91	0,995292686	Breast Neoplasms	Coronary artery disease
62	288	0,995292205	Carcinoma, Renal Cell	Obesity
51	168	0,995292088	Breast Neoplasms	Hepatitis C
211	262	0,995291125	Leukemia, Lymphocytic, Acute	Multiple Myeloma
84	108	0,995290581	Colitis, Ulcerative	Diabetes Mellitus
124	211	0,995290533	Endometriosis	Leukemia, Lymphocytic, Acute
124	262	0,995287792	Endometriosis	Multiple Myeloma
61	91	0,995286221	Carcinoma, Pancreatic Ductal	Coronary artery disease
23	282	0,995285851	Aortic Valve Stenosis	Neuroblastoma
176	385	0,995285826	Hodgkin Disease	Urinary Bladder Neoplasms
8	86	0,995282481	Alzheimer Disease	Colorectal Neoplasms
229	238	0,995281738	Lung Neoplasms	Lymphoma, Large-Cell, Diffuse
238	386	0,99528141	Lymphoma, Large-Cell, Diffuse	Uterine Cervical Neoplasms
153	250	0,995280593	Gout	Melanoma
30	298	0,995278508	Asthma	Osteosarcoma
30	149	0,995278135	Asthma	Glioma
91	346	0,995276751	Coronary artery disease	Salivary Gland Neoplasms
8	222	0,995275683	Alzheimer Disease	Liver Cirrhosis
129	235	0,995274501	Esophageal Neoplasms	Lymphoma
108	149	0,995274063	Diabetes Mellitus	Glioma
110	385	0,995273662	Diabetes Mellitus, Type 2	Urinary Bladder Neoplasms
29	167	0,995272106	Arthritis, Rheumatoid	Hepatitis B
110	308	0,995270775	Diabetes Mellitus, Type 2	Parkinson Disease
165	250	0,99526924	Hematologic Neoplasms	Melanoma

129	282	0,995269012	Esophageal Neoplasms	Neuroblastoma
29	386	0,995267429	Arthritis, Rheumatoid	Uterine Cervical Neoplasms
91	271	0,995265181	Coronary artery disease	Myocardial Infarction
29	61	0,995263108	Arthritis, Rheumatoid	Carcinoma, Pancreatic Ductal
256	323	0,995262649	Metabolic Syndrome X	Pre-Eclampsia
91	292	0,995260003	Coronary artery disease	Osteoarthritis
8	334	0,995258242	Alzheimer Disease	Pulmonary Fibrosis
129	350	0,99525736	Esophageal Neoplasms	Sarcoma, Kaposi
35	250	0,995257193	Autistic Disorder	Melanoma
86	330	0,995256021	Colorectal Neoplasms	Psoriasis
124	292	0,995253069	Endometriosis	Osteoarthritis
29	262	0,995251942	Arthritis, Rheumatoid	Multiple Myeloma
51	230	0,995248878	Breast Neoplasms	Lupus Erythematosus, Systemic
209	302	0,995245891	Leukemia, B-Cell, Chronic	Ovarian Neoplasms
211	266	0,995244629	Leukemia, Lymphocytic, Acute	Muscular Dystrophy, Duchenne
167	329	0,99524451	Hepatitis B	Prostatic Neoplasms
61	288	0,99524322	Carcinoma, Pancreatic Ductal	Obesity
61	384	0,995242646	Carcinoma, Pancreatic Ductal	Tuberculosis, Pulmonary
271	308	0,99524257	Myocardial Infarction	Parkinson Disease
67	384	0,995239967	Cardiomyopathy, Hypertrophic	Tuberculosis, Pulmonary
282	330	0,995238889	Neuroblastoma	Psoriasis
266	365	0,995236744	Muscular Dystrophy, Duchenne	Stomach Neoplasms
168	330	0,995236728	Hepatitis C	Psoriasis
167	384	0,995235916	Hepatitis B	Tuberculosis, Pulmonary
62	176	0,995235186	Carcinoma, Renal Cell	Hodgkin Disease
33	62	0,995235	Atrial Fibrillation	Carcinoma, Renal Cell
271	346	0,995234917	Myocardial Infarction	Salivary Gland Neoplasms
86	329	0,995234336	Colorectal Neoplasms	Prostatic Neoplasms
110	334	0,995234281	Diabetes Mellitus, Type 2	Pulmonary Fibrosis
35	365	0,995232954	Autistic Disorder	Stomach Neoplasms
23	149	0,995232899	Aortic Valve Stenosis	Glioma
5	327	0,995231706	Adrenocortical Carcinoma	Prolactinoma
249	263	0,995230852	Medulloblastoma	Multiple Sclerosis
5	308	0,995228699	Adrenocortical Carcinoma	Parkinson Disease
182	282	0,995227426	Hypertension	Neuroblastoma
167	385	0,995226642	Hepatitis B	Urinary Bladder Neoplasms
149	176	0,995225433	Glioma	Hodgkin Disease
5	29	0,995223845	Adrenocortical Carcinoma	Arthritis, Rheumatoid
58	339	0,995223054	Carcinoma, Hepatocellular	Respiratory Syncytial Virus Infections
282	384	0,995221984	Neuroblastoma	Tuberculosis, Pulmonary
346	365	0,995216649	Salivary Gland Neoplasms	Stomach Neoplasms
91	209	0,995212333	Coronary artery disease	Leukemia, B-Cell, Chronic
8	316	0,995211069	Alzheimer Disease	Pituitary Neoplasms
213	384	0,995208975	Leukemia, Myelocytic, Acute	Tuberculosis, Pulmonary
129	238	0,995207753	Esophageal Neoplasms	Lymphoma, Large-Cell, Diffuse
110	330	0,995206647	Diabetes Mellitus, Type 2	Psoriasis
32	330	0,995205865	Atherosclerosis	Psoriasis
165	282	0,995205381	Hematologic Neoplasms	Neuroblastoma
330	350	0,995204262	Psoriasis	Sarcoma, Kaposi
62	316	0,995204107	Carcinoma, Renal Cell	Pituitary Neoplasms
167	330	0,995202531	Hepatitis B	Psoriasis
222	323	0,995200913	Liver Cirrhosis	Pre-Eclampsia
236	271	0,995200599	Lymphoma, B-Cell	Myocardial Infarction
298	385	0,995198215	Osteosarcoma	Urinary Bladder Neoplasms
249	329	0,995197154	Medulloblastoma	Prostatic Neoplasms
213	236	0,995196928	Leukemia, Myelocytic, Acute	Lymphoma, B-Cell
32	266	0,995196455	Atherosclerosis	Muscular Dystrophy, Duchenne
215	266	0,995194146	Leukemia, Myeloid, Chronic	Muscular Dystrophy, Duchenne
67	292	0,995193746	Cardiomyopathy, Hypertrophic	Osteoarthritis
8	13	0,995189084	Alzheimer Disease	Aneurysm
149	236	0,995188925	Glioma	Lymphoma, B-Cell
13	108	0,995186552	Aneurysm	Diabetes Mellitus
271	329	0,995186364	Myocardial Infarction	Prostatic Neoplasms
108	298	0,99518576	Diabetes Mellitus	Osteosarcoma
29	168	0,995185366	Arthritis, Rheumatoid	Hepatitis C
206	229	0,995183747	Leiomyoma	Lung Neoplasms
124	167	0,995183017	Endometriosis	Hepatitis B
23	61	0,995181589	Aortic Valve Stenosis	Carcinoma, Pancreatic Ductal
29	350	0,995181093	Arthritis, Rheumatoid	Sarcoma, Kaposi

62	292	0,995180808	Carcinoma, Renal Cell	Osteoarthritis
124	182	0,995180379	Endometriosis	Hypertension
168	230	0,9951801	Hepatitis C	Lupus Erythematosus, Systemic
51	288	0,995178825	Breast Neoplasms	Obesity
230	249	0,995178691	Lupus Erythematosus, Systemic	Medulloblastoma
62	215	0,99517803	Carcinoma, Renal Cell	Leukemia, Myeloid, Chronic
211	323	0,995175903	Leukemia, Lymphocytic, Acute	Pre-Eclampsia
262	303	0,995174246	Multiple Myeloma	Pancreatic Neoplasms
29	91	0,995172804	Arthritis, Rheumatoid	Coronary artery disease
213	225	0,995171551	Leukemia, Myelocytic, Acute	Liver Neoplasms
29	33	0,995170978	Arthritis, Rheumatoid	Atrial Fibrillation
84	167	0,995170083	Colitis, Ulcerative	Hepatitis B
5	165	0,995169376	Adrenocortical Carcinoma	Hematologic Neoplasms
108	385	0,995169302	Diabetes Mellitus	Urinary Bladder Neoplasms
13	323	0,995167398	Aneurysm	Pre-Eclampsia
30	386	0,995161792	Asthma	Uterine Cervical Neoplasms
250	308	0,995158765	Melanoma	Parkinson Disease
32	129	0,995158269	Atherosclerosis	Esophageal Neoplasms
8	262	0,995157633	Alzheimer Disease	Multiple Myeloma
129	211	0,995155259	Esophageal Neoplasms	Leukemia, Lymphocytic, Acute
288	339	0,995154587	Obesity	Respiratory Syncytial Virus Infections
13	58	0,995154183	Aneurysm	Carcinoma, Hepatocellular
230	292	0,995153976	Lupus Erythematosus, Systemic	Osteoarthritis
211	282	0,995152322	Leukemia, Lymphocytic, Acute	Neuroblastoma
13	292	0,995150585	Aneurysm	Osteoarthritis
29	211	0,99514835	Arthritis, Rheumatoid	Leukemia, Lymphocytic, Acute
235	330	0,995147846	Lymphoma	Psoriasis
23	110	0,995144698	Aortic Valve Stenosis	Diabetes Mellitus, Type 2
5	222	0,995142803	Adrenocortical Carcinoma	Liver Cirrhosis
8	329	0,995142497	Alzheimer Disease	Prostatic Neoplasms
67	182	0,995141606	Cardiomyopathy, Hypertrophic	Hypertension
91	262	0,995141466	Coronary artery disease	Multiple Myeloma
128	168	0,995140942	Epilepsy	Hepatitis C
249	342	0,995140401	Medulloblastoma	Retinoblastoma
262	263	0,995140073	Multiple Myeloma	Multiple Sclerosis
